# Weight Optimization of Steel Tied-Arch Footbridge

**DOI:** 10.3390/ma19153288

**Published:** 2026-08-03

**Authors:** Damian Sokołowski, Tomasz Wudkiewicz

**Affiliations:** Department of Structural Mechanics, Faculty of Civil Engineering, Architecture & Environmental Engineering, Lodz University of Technology, Al. Politechniki 6, 90-924 Lodz, Poland; tomasz.wudkiewicz@dokt.p.lodz.pl

**Keywords:** steel tied-arch footbridge, parametric optimization, structural optimization, finite element analysis, Eurocode, arch girder, hanger arrangement

## Abstract

**Highlights:**

- Parametric optimization reduced tied-arch footbridge arch-girder weight by 10.7%.
- Arch rise and hanger number were the key drivers of the minimum-weight design.
- A Dynamo–Python workflow automated FEM generation and Eurocode-based verification.
- The optimum solution used a 23.4 m arch rise, 31 hangers, and 6.0 m deck spacing.
- API-based workflows can support early-stage optimization of tied-arch footbridges.
- Eurocode-based sizing helps identify the most influential design variables.
- Hanger arrangement strongly influences force redistribution in the arch girder.
- ULS constraints and discrete section changes shape the minimum-weight solution.

**Abstract:**

This study presents a materials-oriented, code-based parametric optimization of the load-bearing steel tubular arch girder in a tied-arch footbridge inspired by the Father Bernatek Footbridge in Krakow. The objective was to reduce structural steel demand by minimizing the arch-girder weight under Eurocode load combinations with ultimate limit state (ULS) and serviceability limit state (SLS) constraints, while accounting for discrete tubular cross-section changes within a realistic finite element model. A semi-automated workflow linked Autodesk Dynamo, Python scripts, and Autodesk Robot Structural Analysis to generate bridge geometry, build the finite element method (FEM) model, apply code-based loads and combinations, and evaluate structural response using a discrete, non-gradient-based search. A preliminary sensitivity screening was performed for the full set of design parameters, while the final optimization was governed mainly by arch rise, hanger number, and ULS-controlled discrete arch cross-section changes. The optimization reduced the arch-girder weight by 10.7% relative to the reference configuration, from 360.3 × 10^3^ kg to 321.6 × 10^3^ kg, within the adopted design domain. The optimum solution corresponded to an arch rise of 23.4 m, 31 hangers, and a deck spacing of 6.0 m. Hanger arrangement strongly affected force redistribution in the arch girder, while the final optimum was controlled by code-based utilization thresholds. The results show that an application programming interface (API)-driven parametric workflow can support early-stage optimization of tied-arch footbridges under code-based design constraints. The scientific contribution of the study lies not in automating Eurocode verification alone, but in identifying the structural mechanisms that govern the minimum-weight solution, including the interaction between arch rise, hanger arrangement, force redistribution, ULS utilization, and discrete tubular cross-section changes.

## 1. Introduction

Arch bridges combine structural efficiency with strong architectural appeal. Many have become local landmarks, including the Pont du Gard in France, the Rialto Bridge in Venice, and the Sydney Harbour Bridge. In Poland, large arch bridges are reviewed in detail by Siwowski et al. [1]. Biliszczuk summarizes the development of bridge engineering in independent Poland [2]. Standard design principles for steel bridges, including arches, are compiled in [3]. Recent research continues to refine conceptual choices, including the influence of arch position relative to the deck on global structural response [4]. Engineers increasingly use parametric workflows to explore such variants during the early design stages [5].

Tied-arch bridges form an important arch-bridge family. They replace foundation thrust resistance with a tie girder in tension, which enables longer spans and reduces abutment demands. Researchers have proposed minimum-weight sizing rules for steel tied-arch bridges under deflection limits [6]. Others have optimized tied-arch designs under code constraints using metaheuristic search methods [7]. Additional studies have examined long-span truss–arch configurations with different arch-seat arrangements and load paths [8], while experimental investigations of arch-rib systems have improved understanding of ultimate resistance mechanisms [9]. Reliability-based formulations have also been incorporated directly into the optimization process [10,11].

Geometry plays a decisive role in the structural behavior of arch and tied-arch bridges. Arch curvature, rise-to-span ratio, deck shape, and hanger layout all influence structural response. Visual-programming-based finite element method (FEM) workflows combined with genetic algorithms (GA) enable efficient exploration of such design spaces [12]. Genetic algorithm approaches have also been applied to optimize the use of high-performance steel in arch bridges [13]. Multi-objective techniques allow simultaneous optimization of structural components with respect to competing criteria [14], while step-by-step evolutionary procedures provide practical optimization strategies for bridge structures [15]. Pareto-based methods can further improve computational efficiency in multi-objective problems [16].

Although parametric and algorithm-aided workflows have become increasingly common in bridge engineering, existing approaches still show several limitations when applied to practical tied-arch bridge design. General reviews of structural optimization in civil engineering indicate that many studies still rely on simplified structural models, problem-specific objective functions, and optimization strategies that are difficult to transfer directly into code-based engineering practice [17]. Reviews focused specifically on bridge optimization further show that previous studies often differ substantially in terms of objective functions, structural models, optimization algorithms, and computational tools, which makes the direct transfer of such procedures to practical bridge design workflows non-trivial [18]. Algorithm-aided bridge design studies have demonstrated that parametric workflows can reduce modeling effort and support the exploration of alternative structural solutions [19]. Recent reviews of parametric BIM and forward-design approaches in bridge engineering also confirm that parametric modeling can improve design exploration and interoperability, but the full integration of geometry generation, analysis-model creation, structural verification, and optimization remains only partially resolved [20].

Preliminary minimum-weight formulations for tied-arch bridges provide valuable insight into the influence of span, rise, and deflection constraints, but they are often based on simplified design criteria and do not fully reproduce the iterative code-based sizing process required in practical structural design [6]. Metaheuristic approaches, including artificial bee colony algorithms, genetic algorithms, harmony search, and related population-based methods, have demonstrated strong capabilities in exploring nonlinear and discrete design spaces [7,12,13,14]. Surrogate-assisted optimization can reduce the number of expensive structural evaluations and has recently been applied successfully to bridge cost optimization [21]. Nevertheless, surrogate models require additional sampling, training, and validation, and their accuracy must be carefully assessed when discrete cross-section changes, load combinations, and code-based utilization checks govern the objective function. Moreover, recent studies on intelligent bridge design indicate that parametric FEM models can support structural analysis and optimization, but the automatic definition of loads and boundary conditions in complex bridge models is still often limited, and many optimization studies remain focused on selected load cases or specific material systems [22]. Therefore, in FEM-based bridge optimization, the choice of the search strategy should be related not only to global-search capability, but also to computational cost, transparency, reproducibility, and compatibility with engineering verification procedures.

These limitations define the research gap addressed in the present study. Existing bridge optimization tools are often efficient either at the level of geometric exploration or at the level of numerical search, but they less frequently combine parametric modeling, direct FEM generation, automated load definition, load-combination evaluation, and Eurocode-based member verification in one reproducible design loop. This gap is particularly important for steel tied-arch footbridges, where the optimum design is governed not only by global geometry, but also by hanger arrangement, arch stability, discrete tubular cross-section changes, and ULS/SLS utilization. The methodological advancement of this work is therefore the development of a semi-automated API-driven workflow that links Dynamo-based parametric modeling, Python scripting, Autodesk Robot Structural Analysis, and Eurocode-based verification. In contrast to studies focused mainly on abstract optimization algorithms, simplified deflection-based criteria, surrogate response models, or black-box metaheuristic search, the proposed procedure treats optimization as an integrated engineering workflow in which every design variant is automatically generated, analyzed, and verified according to code requirements.

Optimization studies of other long-span systems, including those subjected to spatially varying ground motions, also provide transferable methodological insights [23]. Complementary studies on curved structural systems provide guidance on algorithm selection in geometrically nonlinear problems [24], while construction-stage optimization has been addressed in the context of cable-supported erection processes [25].

Material selection strongly affects stiffness, stability, and self-weight. Studies on high-strength steel composite bridges have shown that optimization of steel members can improve structural efficiency [26]. Ultra-High-Performance Concrete (UHPC) solutions are increasingly incorporated into truss and arch systems to reduce weight while maintaining structural performance [27]. Recent developments include double-deck steel truss arch bridges with lightweight UHPC composite decks that satisfy both static and dynamic requirements [28].

Concrete-Filled Steel Tube (CFST) members offer an attractive alternative for arch girders because they combine a steel tube with a confined concrete core. Design-oriented background on CFST behavior is provided in [29], while experimental studies describe the confinement effect in detail [30]. The axial compression performance relevant to arch ribs has also been quantified [31]. Bridge-scale applications are summarized in [32], and recent studies have used refined full-bridge FEM models to optimize stresses in long-span CFST arch bridges [33]. Fatigue performance and construction-stage optimization remain critical aspects of long-term structural behavior [34,35,36,37,38].

Nonlinear stability remains a fundamental issue in slender arch structures. Field measurements provide realistic data on geometric imperfections in steel tied-arch bridges [39]. Analytical studies have examined in-plane buckling behavior under changing support stiffness [40] and predicted elastoplastic buckling of parabolic arches [41]. Additional research has evaluated nonlinear elastic stability across different rise-to-span ratios [42] and applied performance-based design approaches incorporating inelastic nonlinear analysis [43]. Out-of-plane buckling behavior has also been investigated experimentally and numerically for various arch systems [44,45,46].

The methodological advancement of the present study lies in treating optimization not as an isolated numerical search problem, but as an integrated code-based engineering workflow. In contrast to approaches focused mainly on simplified analytical formulations, abstract benchmark optimization, or black-box metaheuristic search, the proposed procedure combines parametric geometry generation, direct FEM model creation, automatic load definition, load-combination evaluation, and Eurocode-based ULS/SLS member verification within one iterative loop. This integration makes it possible to evaluate each candidate design as a structurally verified bridge variant rather than only as a geometrically feasible or numerically optimal solution. As a result, the workflow provides not only the minimum-weight configuration within the analyzed domain, but also an interpretable identification of the governing design variables and structural constraints controlling the final solution.

The proposed methodology is not limited to the investigated tied-arch footbridge. Since the workflow is based on parameterized geometry, API-based model generation, automated analysis, and code-based verification, it can be adapted to other geometry-sensitive structural systems. Potential applications include steel arch bridges with different hanger layouts, truss arch bridges, CFST arch bridges, cable-supported systems, composite bridge decks, roof structures, and spatial frame or shell structures. In such applications, the general optimization loop can remain unchanged, while the parametric geometry definition, member families, load models, and verification criteria should be modified according to the structural typology and relevant design standards.

Recent work has addressed the preliminary design of minimum-weight steel tied-arch bridges using formulations governed primarily by deflection constraints [6]. In contrast, the present study focuses on a steel tied-arch footbridge and adopts a semi-automated workflow that directly integrates parametric modeling, finite element analysis, and Eurocode-based ultimate limit state (ULS) and serviceability limit state (SLS) verification within a single iterative design loop. Consequently, the objective is not limited to identifying a minimum-weight solution. It also includes determining the governing geometric variables and code-based structural constraints that control arch-girder weight in a realistic footbridge design problem. Therefore, this study aims to develop an application programming interface (API)-driven workflow in Dynamo for the code-based parametric optimization of the load-bearing arch girder in a steel tied-arch footbridge and to identify the structural mechanisms that control weight reduction, with particular emphasis on arch rise, hanger arrangement, force redistribution, ULS utilization, and discrete tubular cross-section changes. In this formulation, Eurocode verification is used as a constraint-checking layer, whereas the research contribution consists in coupling this verification with parametric FEM generation and in interpreting the resulting design-space behavior.

## 2. Materials and Methods

### 2.1. Parametric Model

**Figure 1:**
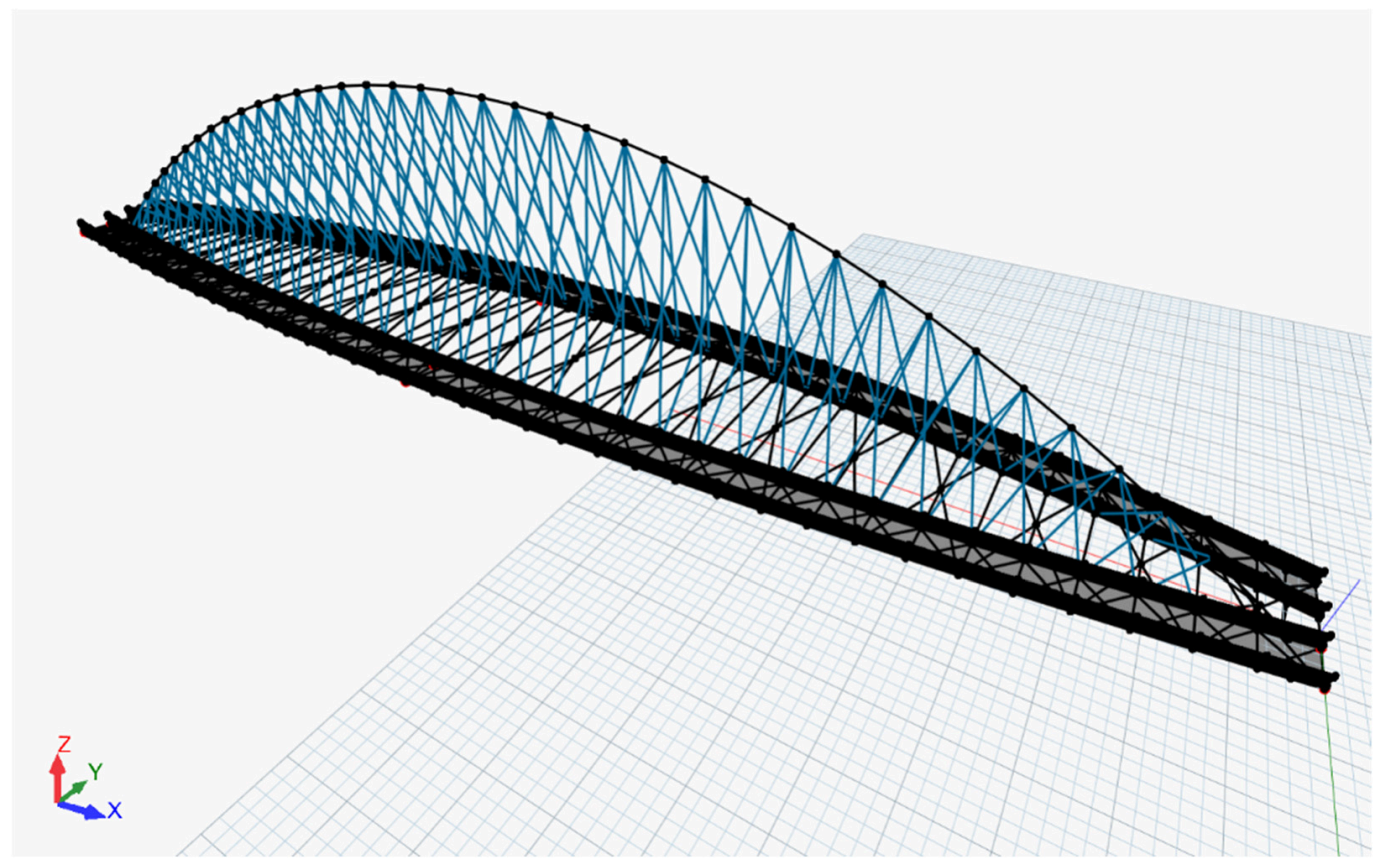
Parametric model of the footbridge developed in Autodesk Dynamo Sandbox.

The parametric model of the footbridge was developed in Autodesk Dynamo Sandbox. Figure 1 presents the structure of the generated bridge model. A similar workflow based on parametric modeling was reported by Kalisz et al. [5].

**Figure 2:**
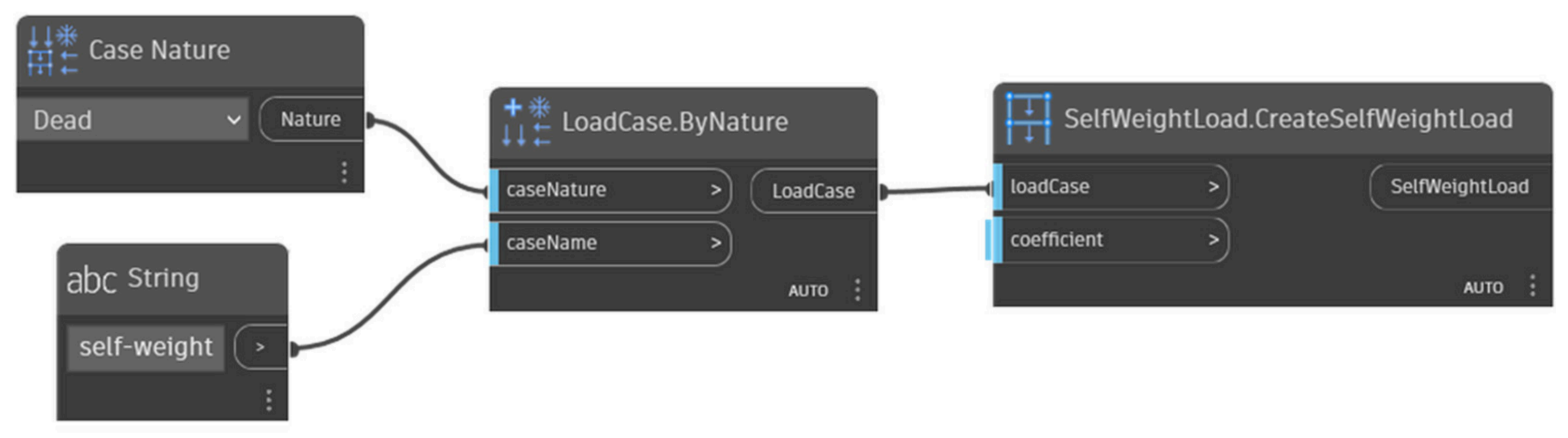
Fragment of the Dynamo script used to define the self-weight load case.

**Figure 3:**
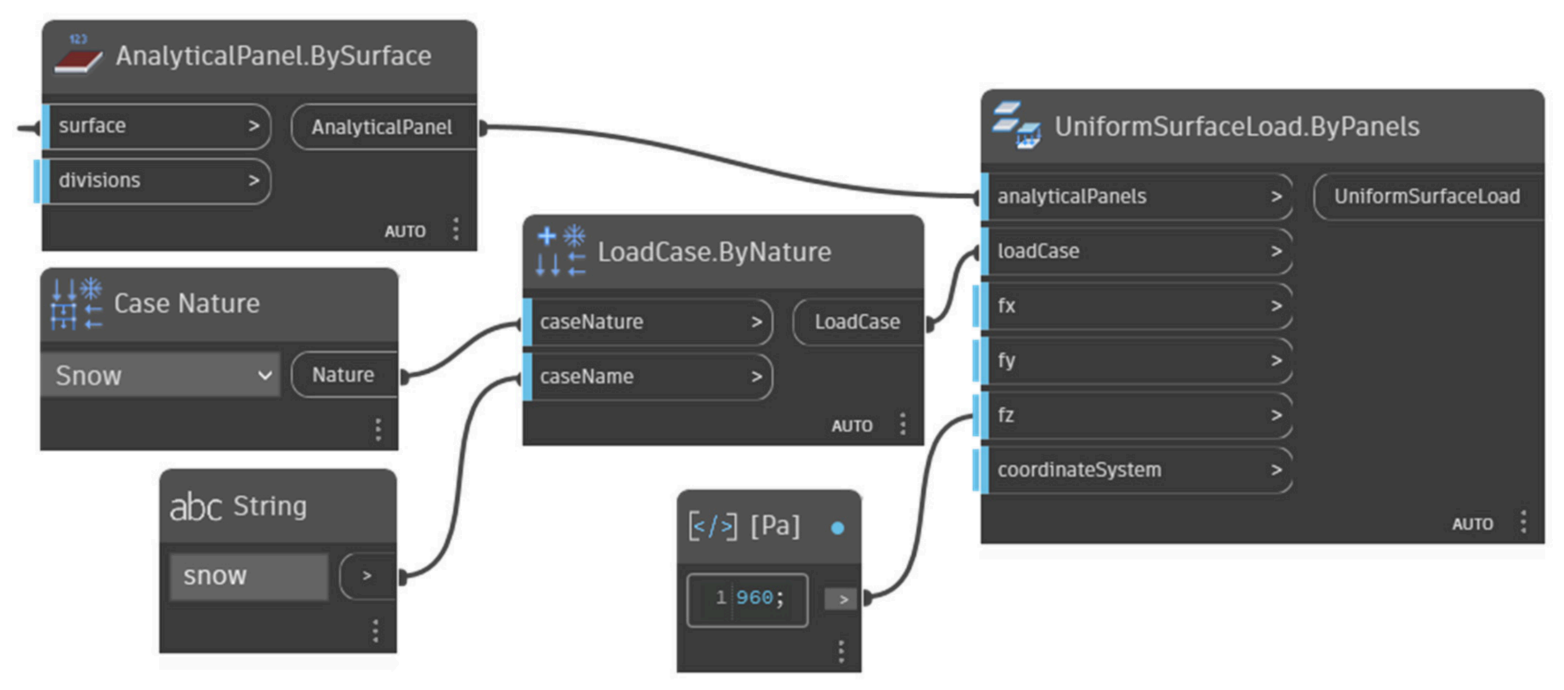
Fragment of the Dynamo script used to define the snow load case.

The modeling workflow combined node-based operations in Dynamo with Python scripts written in CPython 3 and linked to the Robot API. This workflow was used to generate the analytical geometry, assign cross-sections and panel thicknesses, define releases and supports, and introduce the applied loads. Selected parts of the script used to define self-weight and snow load are shown in Figure 2 and Figure 3, respectively. Python routines were additionally used to assign advanced member properties and to automate selected analysis steps, including the implementation of wind loading (Appendix A and Appendix B).

The analytical model represented a tied-arch footbridge composed of an arch girder and two suspended decks connected by hangers. The reference geometry was inspired by the Father Bernatek Footbridge in Krakow. The available global dimensions of the existing bridge were supplemented by the authors’ own field inventory measurements. Since the aim of the present study was not to reproduce the existing structure, but to investigate the material-efficient optimization of a steel tubular arch girder, selected material and cross-section assumptions were adapted to the adopted optimization problem.

The geometry was controlled by a set of design variables listed in Table 1. These variables included the bridge span, arch rise, vertical position of the abutments relative to the deck, number of hangers, deck width, horizontal spacing of the decks, vertical and horizontal curvature of the decks, as well as the diameter and thickness of the arch cross-section. The adopted minimum values, maximum values, and step increments defined the range of the parametric study.

**Figure 4:**
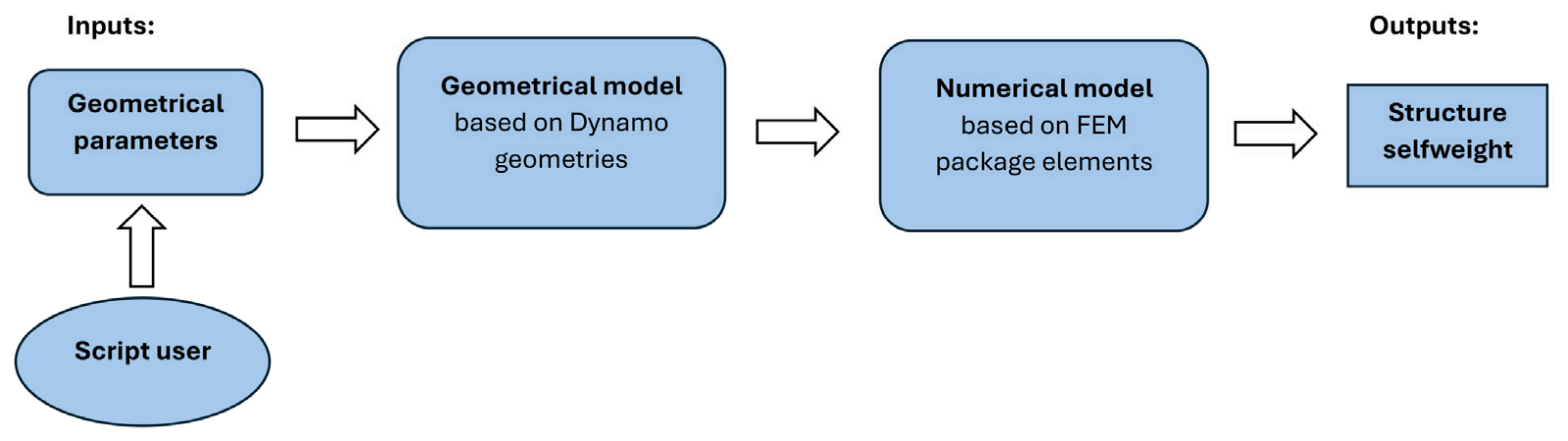
Workflow of the parametric procedure used for the evaluation of structural self-weight.

For each parameter set, the script generated the bridge geometry and transferred the analytical model to Autodesk Robot Structural Analysis. The structure was then analyzed, the relevant member sections were verified according to the adopted ULS/SLS criteria, and the resulting arch girder self-weight was recorded for each iteration and exported automatically to an Excel worksheet. The workflow adopted for the iterative evaluation of self-weight is presented in Figure 4.

The preliminary parametric study indicated that the arch girder self-weight was most strongly affected by arch rise, hanger arrangement, deck spacing, and discrete arch cross-section changes, while the remaining geometric parameters had a secondary influence and were fixed in the subsequent optimization stages.

### 2.2. Numerical Model and Structural Verification

**Figure 5:**
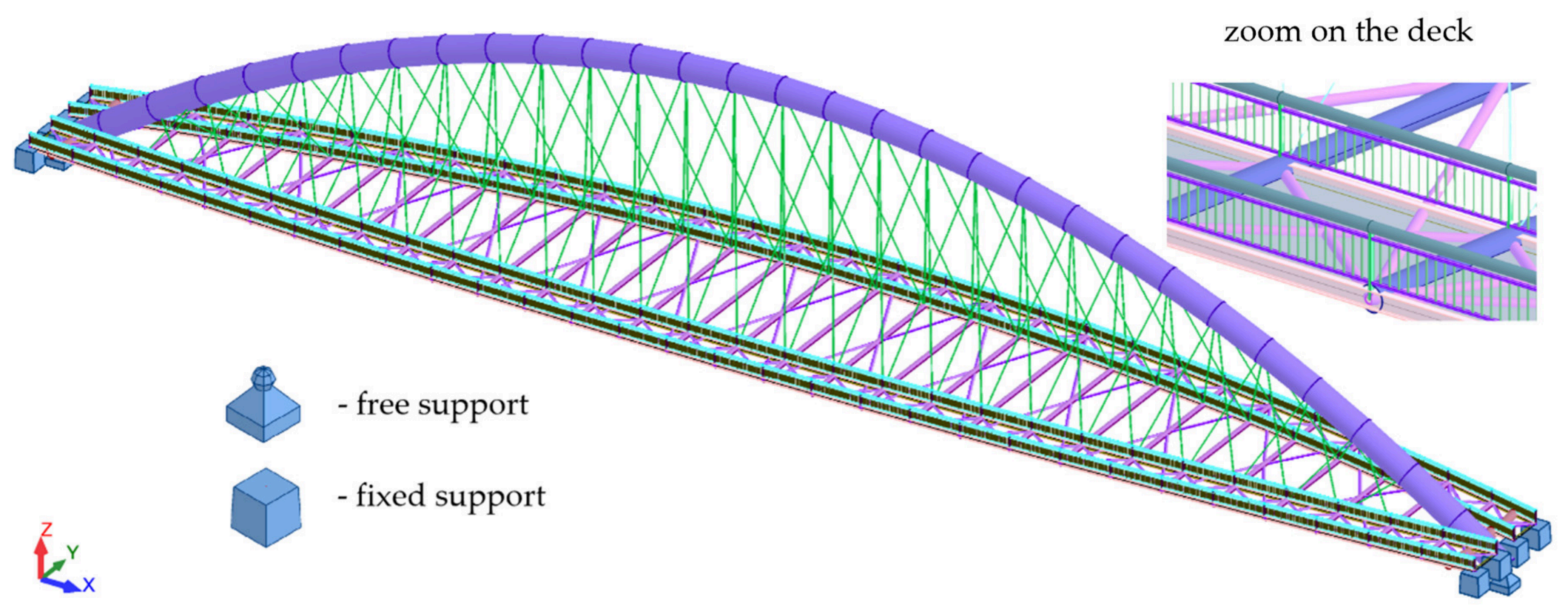
Finite element model of the footbridge used for structural analysis. The colors correspond to the structural components listed in Table 2.

The numerical model of the footbridge, shown in Figure 5, consisted of approximately 3800 beam elements and 5800 shell elements. The shell elements were discretized using the Coons method. A geometrically non-linear analysis was adopted to account for the negligible compressive stiffness of the hangers. The structural calculations and code verification were carried out in Autodesk Robot Structural Analysis.

The structural elements included in the finite element model, together with their corresponding cross-sections, are summarized in Table 2. They are also distinguished by colors in Figure 5. All steel members were assigned S460 M/ML steel. This grade was selected because it provides high strength, good weldability, and sufficient impact toughness at low temperatures, down to −50 °C.

The arch rib was supported by two simple supports, whereas the footbridge decks were fixed at both ends. The abutments were not represented as separate structural components in the numerical model, because their influence on the global response of the analyzed single-span system was considered negligible. The connection between the arch and the decks was modeled using steel hangers. Hinge–hinge releases were introduced at the hanger ends, with torsional rotation about the member axis restrained. The hangers were modeled as tension-only members; therefore, their compressive stiffness was ignored. Additional modeling assumptions were introduced for the X-bracing system, for which only axial tensile and compressive stiffness was considered, while flexural stiffness was neglected.

The load model included self-weight, snow load, live load, and wind load. Self-weight was generated automatically from the structural model. Snow load was applied as a uniformly distributed load on the decks with a characteristic value of 0.96 kN/m^2^, in accordance with Eurocode provisions. The live load was taken as 5.0 kN/m^2^ to represent pedestrian and cyclist traffic. Wind action was introduced on the basis of a wind load simulation including fluid–structure interaction carried out in the dedicated analysis module. The basic wind velocity was assumed as 22 m/s, in accordance with the Eurocode.

The structure was verified using normative ULS and SLS load combinations with the corresponding partial and combination factors. In total, more than 600 load combinations were generated and evaluated during the optimization process, including approximately 400 ULS combinations and 200 SLS combinations. The ULS combinations were based on the Eurocode formulation using alternative expressions 6.10a and 6.10b, in which different actions may become governing depending on the checked structural response. Consequently, the generated set included combinations with different leading variable actions and accompanying actions, such as pedestrian live load, snow load, and wind action, each introduced with the appropriate partial and combination factors. Some load cases were also mutually exclusive or direction-dependent, especially for wind action, which made manual identification of the governing case unreliable.

This extensive load-combination set was essential because the most unfavorable response of the arch girder could not be identified from a single simple load case or from one arbitrarily selected load arrangement. Instead, each design variant had to be checked against a broad set of possible Eurocode-compliant loading scenarios, including different dominant actions, reduced accompanying actions, asymmetric deck loading, climatic actions, and wind directions. The automatic generation and evaluation of these combinations therefore formed an important part of the proposed workflow and reduced the risk of underestimating the governing ULS or SLS demand.

**Figure 6:**
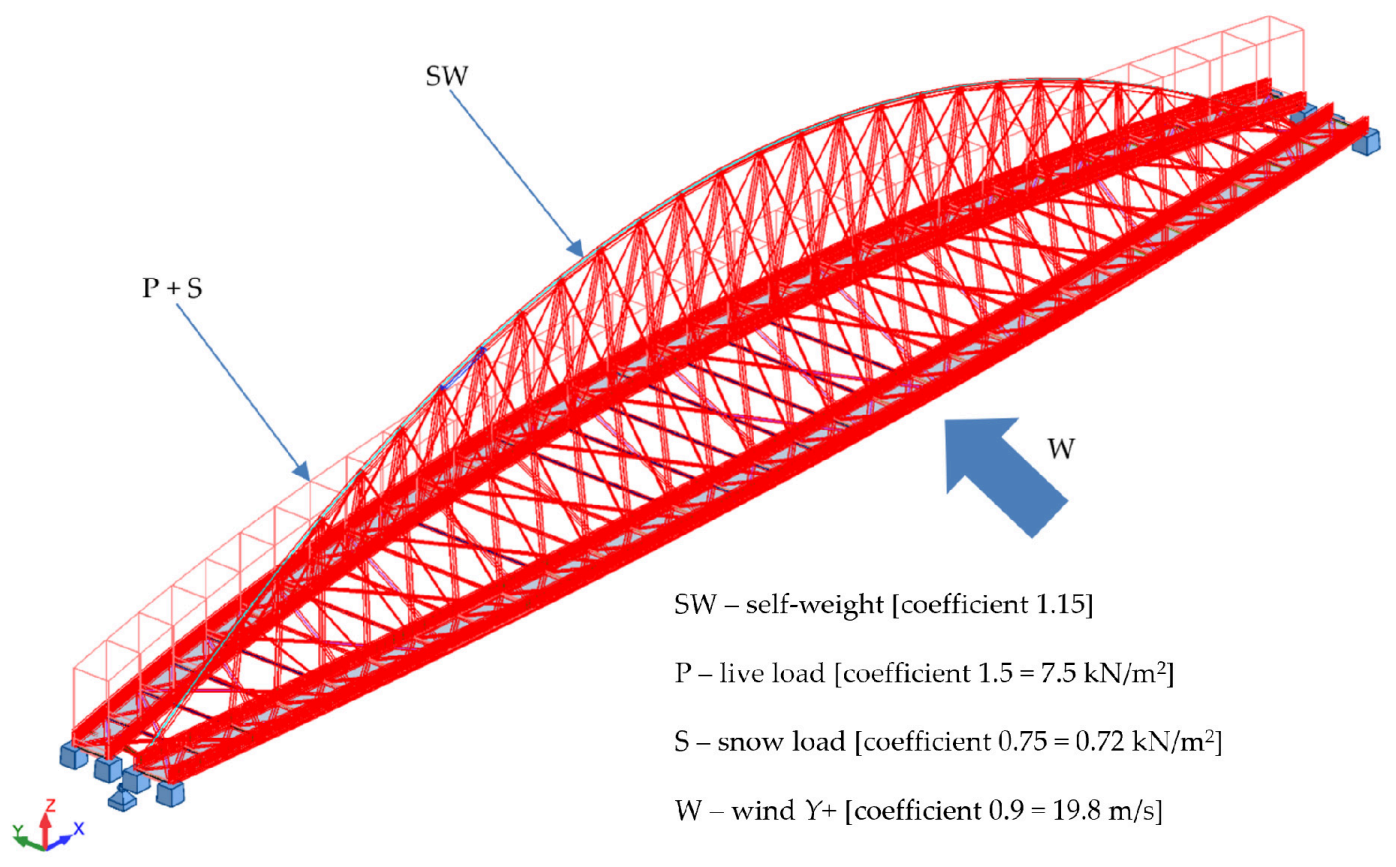
Load arrangement for the governing ULS combination SGN/176 in the optimized configuration.

For the optimized configuration, the governing ultimate limit state (ULS) case was load combination No. 185, denoted as SGN/176 in the calculation output. The number 185 refers to the Robot output case number, whereas SGN/176 denotes the corresponding ULS combination label. The corresponding load arrangement is shown in Figure 6. This combination consisted of permanent action due to self-weight with a partial factor of 1.15, pedestrian live load applied only to the right deck with a factor of 1.50, snow load applied only to the right deck with a factor of 0.75, and automatically generated wind action in the +*Y* direction with a factor of 0.90.

All structural members were verified for the ultimate limit state (ULS) and serviceability limit state (SLS). The arch was treated as the governing load-bearing member, and its cross-section was adjusted in each iteration to minimize structural self-weight while satisfying code requirements.

The arch element was dimensioned according to PN-EN 1993-1-1:2006/NA:2010/A1:2014 [47]. The in-plane and out-of-plane buckling length factors were defined manually on the basis of PN-EN 1993-2 [48]. The out-of-plane buckling factor was determined from the following expression [48]:

*β_z_* = *β*_1_*β*_2_ (1)

where *β*_1_ is a factor dependent on the ratio *f/L* (*f* is the arch rise and *L* is the support spacing) and on the second moment of area *I_z_*. According to Table D.6 in PN-EN 1993-2 [48], *β*_1_ was adopted in the range of 0.54–0.65. The factor *β*_2_ depends on the method of load application to the arch. According to Table D.7 in PN-EN 1993-2 [48], *β*_2_ = 0.65 was adopted. As a result, the final out-of-plane buckling factor ranged from *β_z_* = 0.35 to 0.42. The in-plane buckling factor was adopted from Table D.4 in PN-EN 1993-2 [48] and ranged from *β_y_* = 1.01 to 1.04. To illustrate the influence of arch geometry on the adopted buckling parameters, the variation in the in-plane *β_y_* and out-of-plane *β_z_* buckling coefficients was additionally evaluated as a function of the arch rise-to-span ratio *f*/*L*. This analysis was based on the coefficient ranges adopted from PN-EN 1993-2 and was used to verify how changes in arch rise affect the buckling-length assumptions applied in the ULS verification of the arch girder.

**Figure 7:**
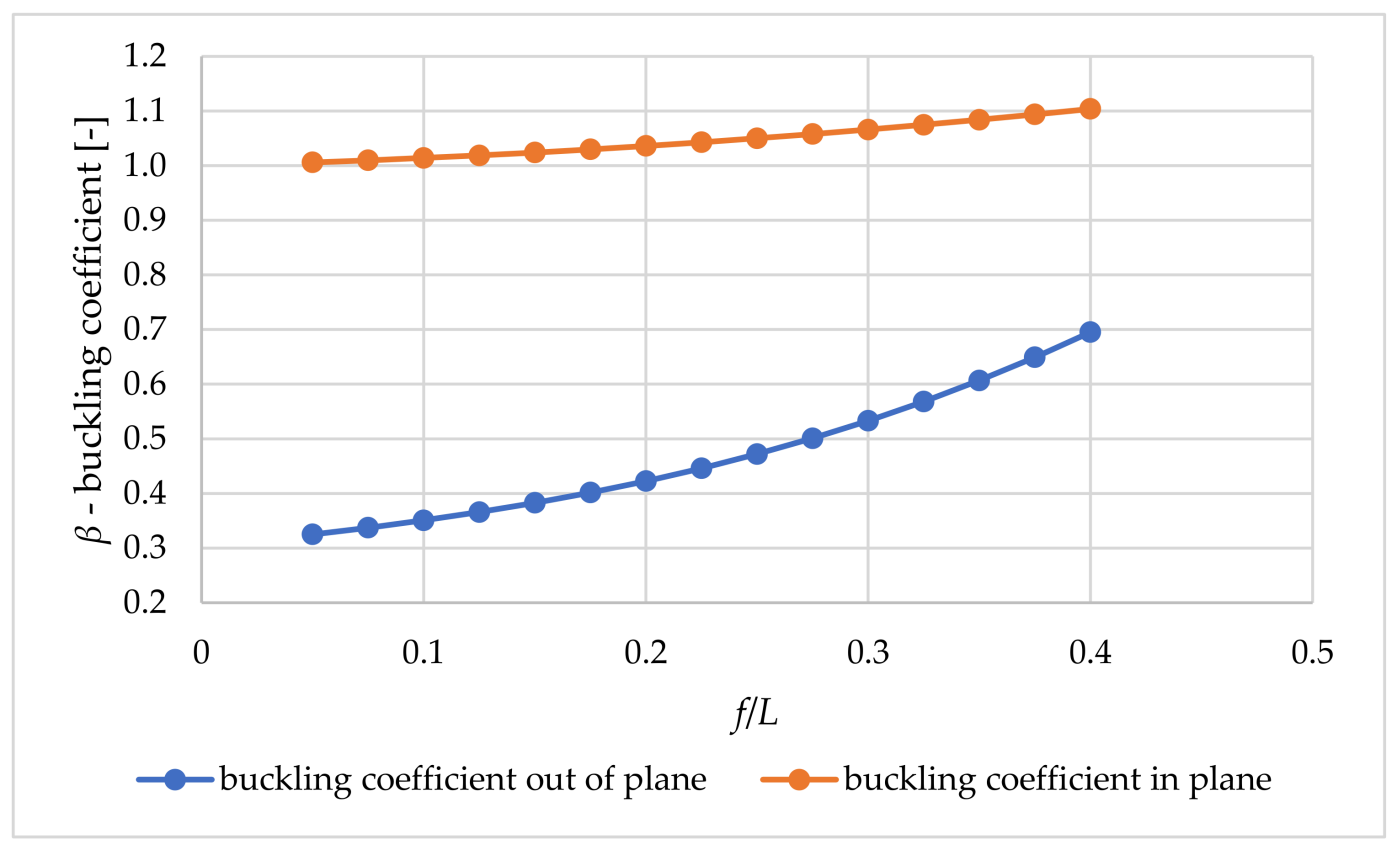
Buckling coefficients as a function of the arch rise-to-span ratio *f*/*L*.

Figure 7 presents the adopted in-plane and out-of-plane buckling coefficients as functions of the arch rise-to-span ratio *f*/*L*. The two curves show the sensitivity of the in-plane coefficient *β_y_* and the out-of-plane coefficient *β_z_* to changes in arch geometry.

As shown in Figure 7, the out-of-plane buckling coefficient *β_z_* was more sensitive to the rise-to-span ratio than the in-plane coefficient *β_y_*. Over the wider *f*/*L* range shown in Figure 7, *β_z_* increased from approximately 0.32–0.35 for low-rise arches to approximately 0.70 for the highest *f*/*L* values considered, whereas the values used in the final optimization range remained considerably narrower. The in-plane coefficient *β_y_* increased only moderately, from approximately 1.00 to 1.10. For the optimized configuration, with an arch rise of 23.4 m and a span of 143 m, the ratio *f*/*L* was approximately 0.164. This corresponded to *β_z_* of about 0.39–0.40 and *β_y_* of about 1.03. For the reference configuration, with an arch rise of 15.0 m, *f*/*L* was approximately 0.105, giving lower corresponding buckling coefficients. Therefore, increasing the arch rise affected not only the global geometry and force redistribution, but also the buckling-length assumptions used in the code-based verification.

The stronger variation in *β_z_* indicates that out-of-plane stability assumptions are more sensitive to changes in arch rise than in-plane buckling assumptions. This is relevant for the present optimization because the final arch cross-section was governed mainly by ULS utilization and discrete section changes. Consequently, even moderate changes in the adopted buckling coefficients could contribute to threshold effects in the required arch cross-section and, therefore, to the observed stepwise variation in arch girder weight.

The governing ULS condition for the arch member was the interaction of axial compression *N_Ed_* and bending moments about both principal axes *M_y_*_,*Ed*_ and *M_z_*_,*Ed*_. The verification was performed using the following expression from [47]:

(2) $$\frac{N_{\mathrm{Ed}}}{\frac{\chi_{y}N_{\mathrm{Rk}}}{\gamma_{M1}}}+k_{\mathrm{yy}}\frac{M_{y,\mathrm{Ed}}\;+\;\Delta M_{y,\mathrm{Ed}}}{\chi_{\mathrm{LT}}\frac{M_{y,\mathrm{Rk}}}{\gamma_{M1}}}+k_{\mathrm{yz}}\frac{M_{z,\mathrm{Ed}}+\Delta M_{z,\mathrm{Ed}}}{\frac{M_{z,\mathrm{Rk}}}{\gamma_{M1}}}\;\leq \;1$$

where *N_Ed_* is the design value of the compressive force; *M_y_*_,*Ed*_, and *M_z_*_,*Ed*_ are the design bending moments about the *y*-*y* and *z*-*z* axes, respectively; Δ*M_y_*_,*Ed*_ and Δ*M_z_*_,*Ed*_ are the additional moments caused by the shift in the centroid in a Class 4 cross-section; *χ_y_* is the flexural buckling factor about the *y*-*y* axis and *χ_LT_* is the lateral–torsional buckling reduction factor; *k_yy_*, *k_yz_* are the interaction factors.

The highest utilization of the arch cross-section was observed at approx. 0.98 *L* along the arch, where *L* denotes the arch length. This section corresponds to the region where the decks were connected to the arch element. The dominant contribution to the utilization ratio was the compressive force *N_Ed_*, which accounted for nearly 66% of the total utilization in each optimization step. The second largest contribution resulted from the bending moment *M_z_*_,*Ed*_, with a utilization of approximately 33%. The contribution of the bending moment *M_y_*_,*Ed*_ was negligible, and the ratio *M_y_*_,*Ed*_/*M_z_*_,*Ed*_ remained below 3% throughout the analyzed cases.

On this basis, the effect of *M_y_*_,*Ed*_ was neglected in the subsequent optimization calculations in order to reduce computational effort. This simplification did not affect the governing resistance condition of the arch girder. In the serviceability limit state, the maximum allowable girder deflection was conservatively limited to *L*/350. The SLS criterion was satisfied in all analyzed iterations and did not govern the design. It should be noted, however, that this serviceability verification was limited to static deflection and did not include pedestrian-induced vibration, comfort criteria, or human–structure interaction effects.

### 2.3. Optimization Procedure

The optimization procedure aimed to minimize the self-weight of the load-bearing arch while satisfying the Eurocode requirements for the ultimate limit state (ULS) and serviceability limit state (SLS), as well as the prescribed bounds of the design variables defined in Table 1. The remaining structural components, including the deck system, bracing, railing, and secondary members, were included in the FEM model and participated in load transfer. These components were assigned the relevant material, cross-section, boundary, and verification parameters and were checked with respect to the applicable ULS and SLS requirements. However, in the subsequent parametric optimization loop, their cross-sections were kept fixed and were not treated as independent objective components. Therefore, the optimization should be interpreted as an arch-girder optimization performed within a complete code-verified FEM model of the tied-arch footbridge, rather than as an isolated arch-only calculation or as a full-bridge optimum. Similar optimization problems for arch-type structures have been reported in the literature [6,10], while alternative optimization approaches were discussed in [7,14,49].

The optimization procedure was preceded by a preliminary sensitivity screening of the full set of design parameters listed in Table 1. This screening was used to identify the variables with the strongest influence on the arch girder self-weight and to fix parameters with secondary influence. The subsequent optimization stages focused mainly on arch rise and hanger number, while deck spacing and vertical deck curvature were assessed and fixed during the preliminary stages. The total bridge span was kept constant at 143 m. For each parameter set, the geometry of the structure was generated automatically, converted into an analytical finite element model, and analyzed under the full set of design actions and code-based combinations. An inner design loop was included in each iteration to update the arch girder cross-section so that the minimum arch girder mass satisfying the code requirements could be obtained. The hangers were selected separately on the basis of manufacturer recommendations and the axial forces obtained from the structural analysis.

The arch cross-section was treated as a discrete design variable rather than as a continuously variable parameter. The outside diameter was varied in 50 mm increments within the range of 2.00–3.00 m, while the wall thickness was varied in 5 mm increments within the range of 30–45 mm. This discretization was adopted to represent practical nominal section selection. The adopted range is consistent with European specifications for hot-finished circular hollow sections, which include outside diameters up to 2500 mm and wall thicknesses up to 120 mm [50]. For project-specific large tubular arch ribs, welded sections fabricated from hot-rolled plates may also be considered; therefore, the plate-thickness tolerances specified in EN 10029:2010 were also taken into account [51]. A detailed explanation of the influence of this discrete section selection on the stepwise variation in arch weight is provided in the Section 4.

**Figure 8:**
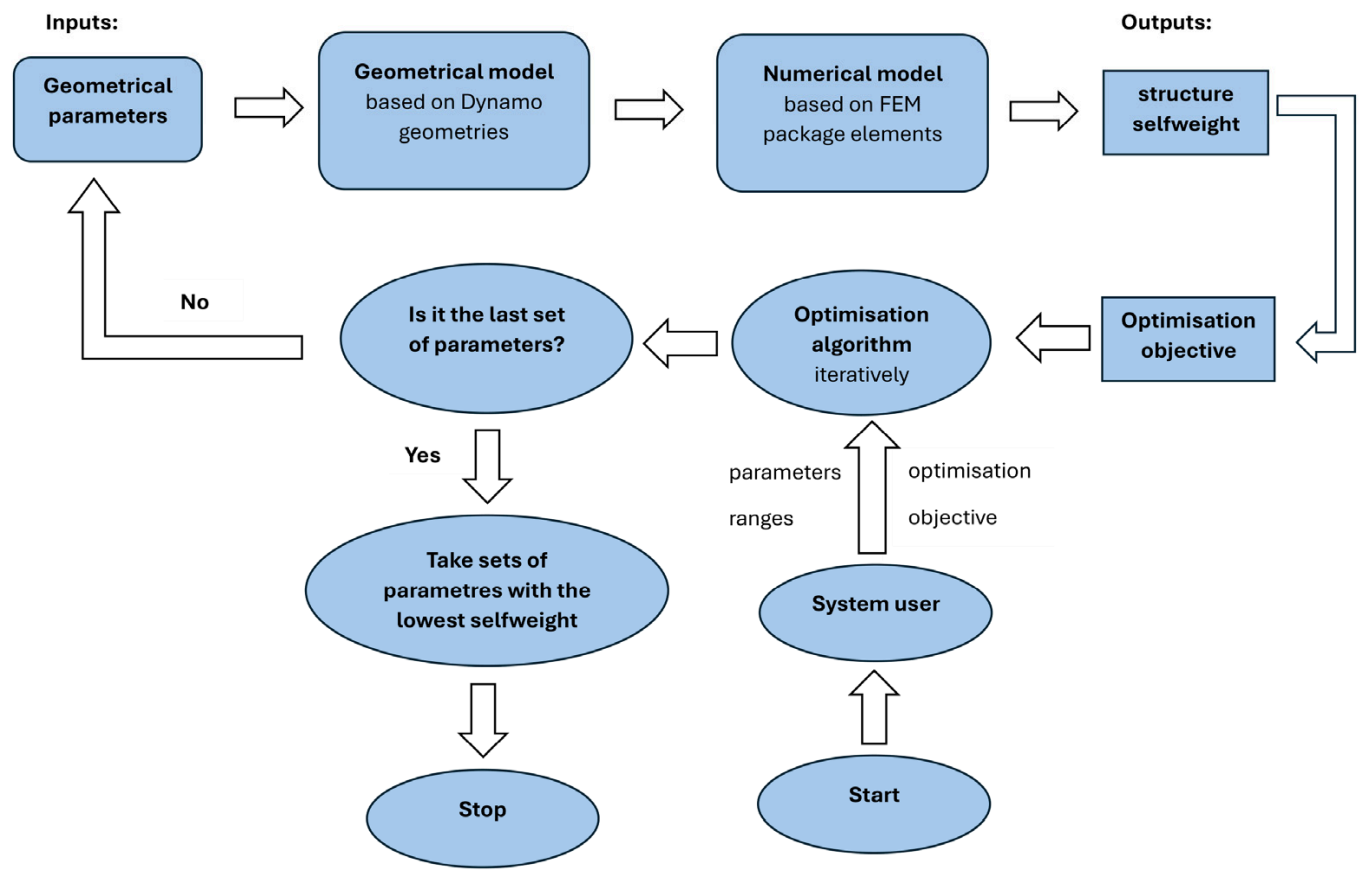
Workflow of the optimization procedure used to minimize the self-weight of the arch girder.

The optimization workflow is presented in Figure 8. The process starts from a user-defined set of geometrical parameters and generates the corresponding parametric geometry. This geometry is then transformed into an analytical model used for finite element analysis. The arch girder self-weight is extracted as the objective function, while the remaining structural members are retained in the FEM model to capture load transfer and code-based verification. The algorithm then updates the parameter set iteratively and repeats the analysis until all predefined cases have been evaluated. The final output consists of the parameter sets that yield the lowest self-weight while satisfying all imposed design constraints.

The search strategy was implemented using progressive domain reduction and consisted of the following stages:

1. Initial screening (coarse sampling).

A preliminary evaluation was performed using the minimum, maximum, and mean values of the governing variables. This stage provided an initial sensitivity assessment and identified the most promising regions of the design space.

2. Deck spacing assessment.

The influence of the horizontal spacing of the decks on the self-weight of the arch member was evaluated using a spacing increment of 0.1 m in the range of 5.5–6.5 m, while the remaining parameters were kept unchanged. The final value of 6.0 m was selected on the basis of both structural response and geometrical admissibility, as discussed in Section 3.

3. Coarse exploration of the design space.

The design space was then explored using a coarse resolution, with an arch-height increment of 3.0 m and a hanger-number increment of 2. This stage enabled identification of the parameter regions associated with lower structural self-weight.

4. Refined arch-height domain.

The arch-height domain was narrowed to 18.0–24.0 m and resampled using a finer increment of 1.0 m. The remaining assumptions were kept unchanged to preserve comparability between successive evaluations.

5. Local probing around candidate optima.

Two candidate arch heights were examined locally within a neighborhood of ±1.0 m using a step of 0.1 m in order to identify the minimum-weight solution more precisely.

Each optimization step included automatic generation of the structural geometry, finite element discretization with the assigned material and code-related parameters, simulation of wind action, generation of load cases and combinations, execution of the numerical analysis, and verification of the ULS and SLS conditions for the arch element. The complete optimization procedure comprised 427 iterations. The final refinement stage, involved 63 iterations and required approximately 7 h of computational time. Considering all optimization stages, the total computational time was approximately 45 h. This semi-automated workflow enabled a systematic exploration of the assumed design space and the identification of the minimum-mass solution that satisfied all structural and normative constraints.

## 3. Results

**Figure 9:**
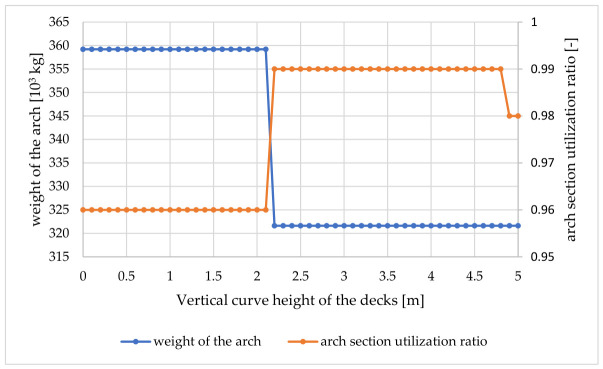
Influence of the vertical curve height of the decks on arch-girder weight and maximum arch section utilization ratio.

The results are first presented in terms of a preliminary sensitivity screening carried out over the parameter ranges defined in Table 1. This stage was used to identify the variables that most strongly influenced the self-weight of the load-bearing arch girder and to determine which parameters could be fixed in the subsequent optimization stages. Apart from the bridge span, the most influential variables were the arch rise, the number of hangers, and the horizontal deck spacing. The remaining geometric parameters had a secondary influence on the arch-girder weight and were therefore fixed once no further beneficial effect was observed. The investigated parameter ranges and increments are reported in Table 1, while the adopted final values and the stage-by-stage evolution of the main optimization variables are summarized in Table 3. As a representative example of the preliminary screening of secondary variables, Figure 9 presents the influence of the vertical curve height of the decks on the arch-girder weight and maximum arch section utilization ratio.

As shown in Figure 9, the response of the arch girder to changes in vertical deck curvature was clearly stepwise. For vertical curve heights up to approximately 2.1 m, the required arch-girder weight remained nearly constant at about 359 × 10^3^ kg, with a utilization ratio of approximately 0.96. When the vertical curve height reached approximately 2.2 m, the required arch cross-section changed, reducing the arch-girder weight to about 322 × 10^3^ kg. Further increases in vertical deck curvature did not produce any additional reduction in arch girder weight, while the utilization ratio remained close to 0.99 over most of the investigated range. Therefore, increasing the vertical curve height beyond approximately 2.2 m was not beneficial for the adopted optimization objective. Moreover, larger vertical curvature increases the length of longitudinal deck members and may therefore increase the weight of the supporting deck structure. Based on these observations, a vertical curve height of 2.2 m was selected and kept constant throughout the subsequent optimization stages.

The vertical deck curvature study was used as a representative example of the preliminary sensitivity checks performed for variables with secondary influence. Similar checks were carried out for the remaining geometric parameters. When a parameter did not produce any further reduction in arch-girder weight or meaningful change in the utilization ratio, its value was fixed in the subsequent optimization stages. The complete set of individual plots and all 427 analyzed combinations is not included in the main text for clarity and brevity, as these additional data would not change the interpretation of the governing design variables. Instead, the investigated parameter ranges, increments, and adopted final values are summarized in Table 1, while the main stage-by-stage optimization results are reported in Table 3.

**Figure 10:**
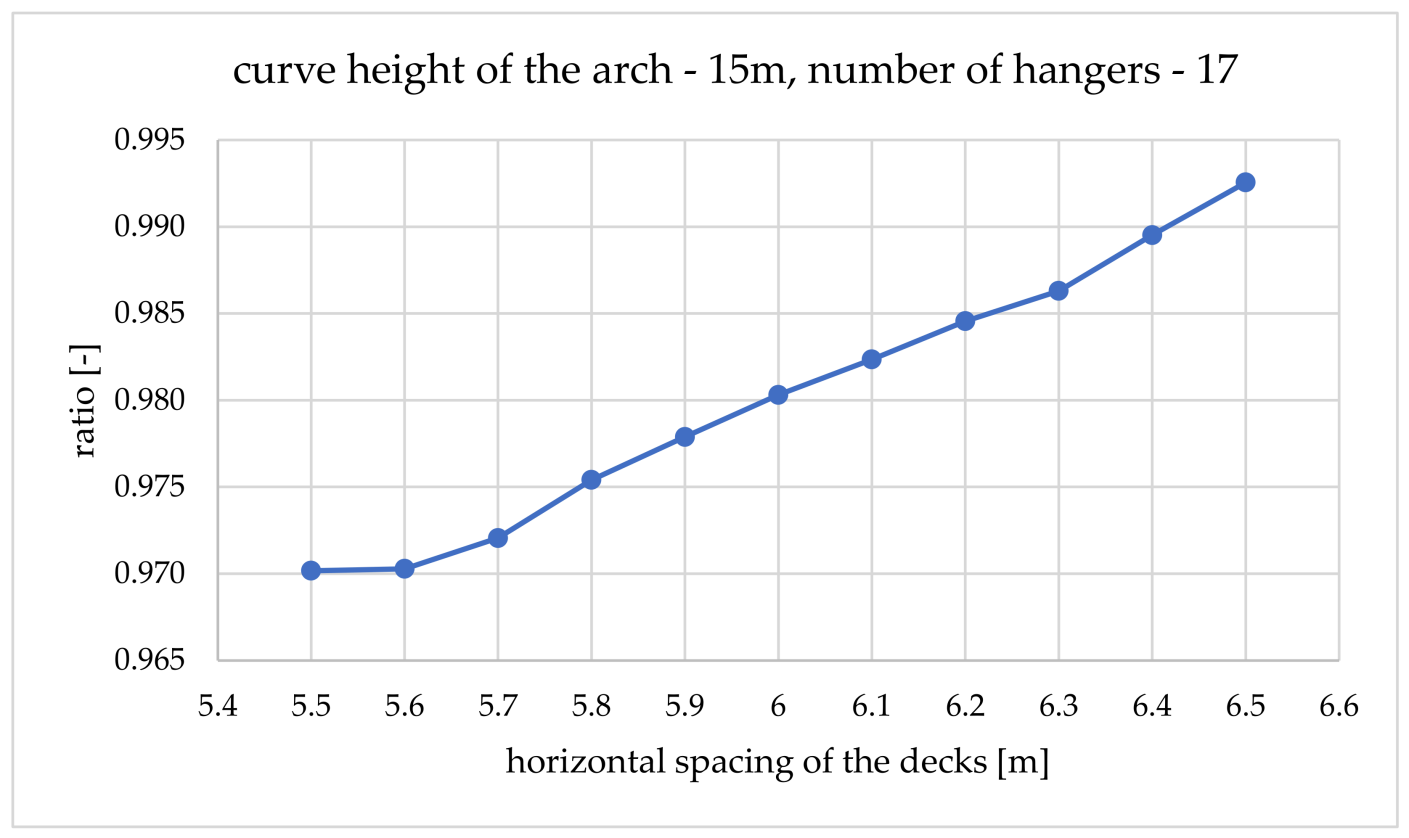
Utilization ratio of the arch girder as a function of deck spacing.

The subsequent optimization stages showed that increasing the horizontal spacing of the decks had an unfavorable effect on the structural response of the load-bearing arch. As shown in Figure 10, the utilization ratio increased progressively with deck spacing for the reference case with a 15.0 m arch rise and 17 hangers. The utilization ratio was calculated using Equation (2) for the most unfavorable load combination at the most highly utilized arch section. This trend indicates that wider deck spacing increased the structural demand in the arch girder without improving its performance. For this reason, deck spacing was not treated as a governing optimization variable in the subsequent stages, and a value of 6.0 m was adopted for the remaining analyses. Although Figure 10 shows that the lowest utilization ratio was obtained for deck spacings of approximately 5.5–5.6 m, the improvement relative to the 6.0 m spacing was marginal, approximately 1%, and did not lead to any further reduction in the required arch girder weight. Therefore, from the structural point of view, reducing the spacing below 6.0 m did not provide a meaningful benefit for the adopted optimization objective. The adoption of 6.0 m was also governed by geometrical constraints. The spacing values were measured between the centerlines of the two footbridge decks. For spacings below 6.0 m, the wider deck zones near the bridge ends began to intersect, which would reduce the available clearance and result in an inadmissible bridge geometry. Consequently, a deck spacing of 6.0 m was selected as the smallest geometrically acceptable value that preserved nearly the same structural performance as the slightly narrower configurations.

**Figure 11:**
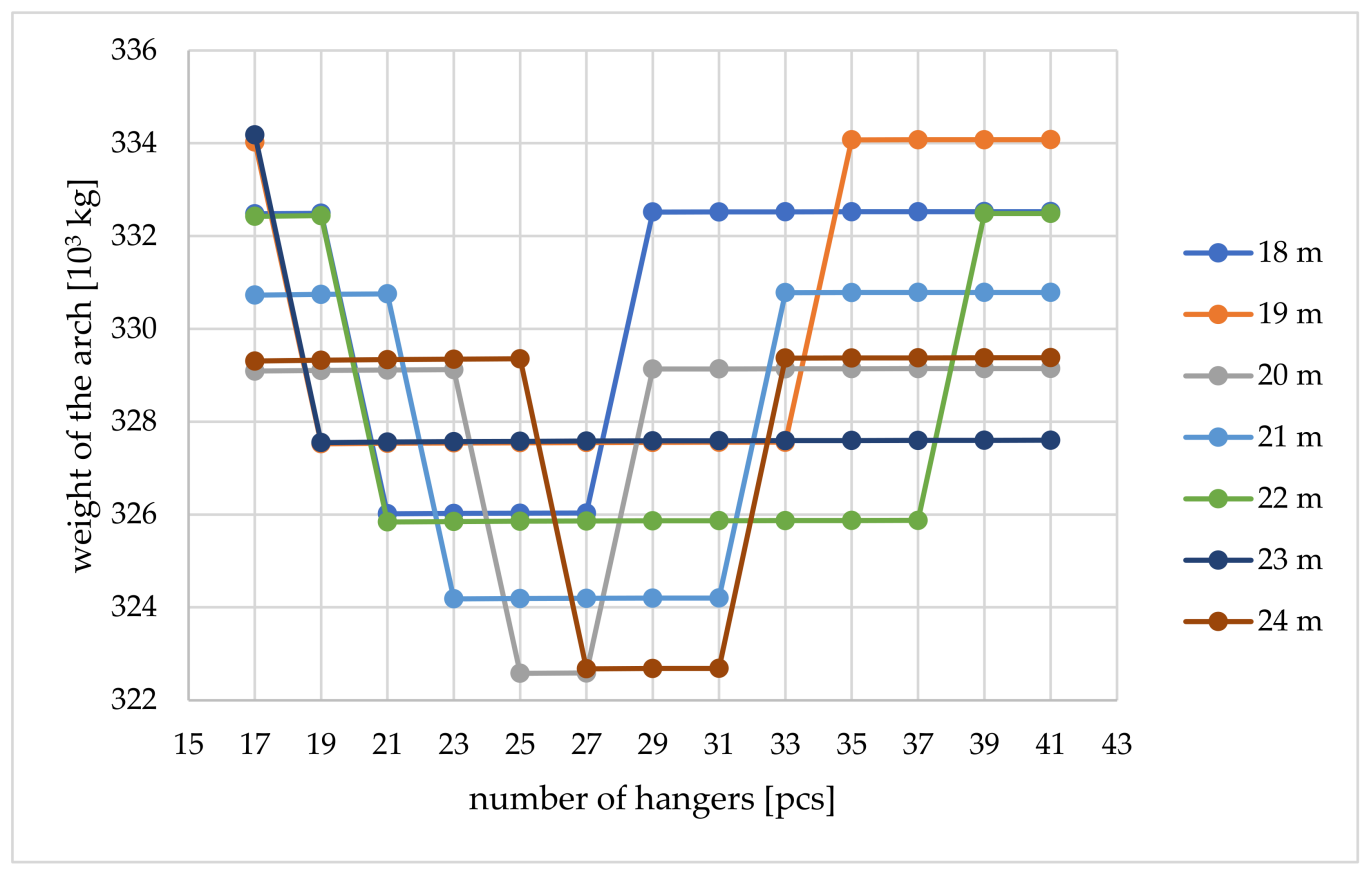
Arch girder self-weight as a function of the number of hangers for different arch rises.

The results further demonstrated that the number of hangers had a direct influence on the self-weight of the arch element and that this influence depended on the arch rise. Figure 11 shows that the minimum self-weight was obtained for different hanger arrangements depending on the arch geometry. For lower arch rises, the optimum was reached with fewer hangers, whereas for higher arch rises the optimum shifted toward denser hanger arrangements. Across the analyzed range, the most favorable solutions were consistently obtained for approximately 25–31 hangers. This confirms that the optimum configuration resulted from the interaction between arch geometry and hanger density rather than from either parameter considered separately.

Additional hangers also introduce extra material mass and require additional anchorages, connection plates, local reinforcement, fabrication operations, corrosion protection, inspection, and installation work. These effects were not included in the objective function, which was limited to the self-weight of the load-bearing arch girder. Although the additional mass of a single hanger is small relative to the arch girder weight, the associated fabrication, installation, and maintenance costs may not be negligible. Therefore, configurations with fewer hangers and comparable arch-girder weight may be preferable from the perspective of total construction cost.

A further result visible in Figure 11 is the stepwise character of the self-weight curves. The self-weight did not change smoothly over the full range of parameters, but instead formed discrete levels for several combinations of arch rise and hanger number. This behavior indicates that the optimization outcome was strongly affected by changes in the required cross-section of the arch member. The largest reductions in self-weight occurred when a given parameter combination allowed the girder to satisfy the ultimate limit state with a lighter cross-section. Within the locally favorable range, additional increases in the number of hangers produced only minor changes in the objective function.

**Figure 12:**
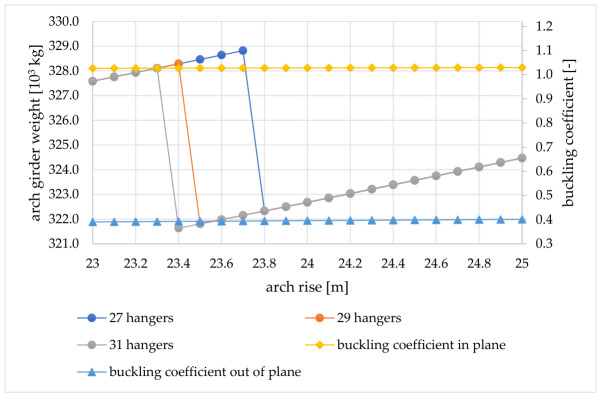
Local refinement of the optimum solution based on arch rise and number of hangers, with adopted in-plane and out-of-plane buckling coefficients.

The local refinement of the search domain identified the final optimum solution, which is presented in Figure 12; it visualizes stage 5 of the optimization. The minimum self-weight of the load-bearing arch was obtained for an arch rise of 23.4 m and 31 hangers. For this configuration, the arch weight was approximately 321.6 × 10^3^ kg. The neighboring solutions with 27 and 29 hangers also produced low self-weight values, but they remained slightly less favorable. The results therefore indicate that the optimum solution was located within a narrow range of arch rises and hanger numbers, and that small deviations from this range led to a measurable increase in structural mass.

Figure 12 also presents the in-plane and out-of-plane buckling coefficients adopted for the corresponding arch-rise range. Within the local refinement interval, the in-plane buckling coefficient remained practically constant, close to 1.0, while the out-of-plane coefficient increased only slightly, from approximately 0.39 to 0.40. This confirms that, in the final search range, changes in the buckling coefficients had a secondary influence compared with the effects of hanger arrangement and discrete arch cross-section selection. Thus, the observed local minimum was governed mainly by the interaction between arch rise, number of hangers, ULS utilization, and the threshold at which a lighter tubular cross-section could satisfy the verification criteria.

Table 3 summarizes the evolution of the main design parameters during the consecutive optimization stages and compares the optimized configuration with the reference structure. The reference configuration was characterized by a 15.0 m arch rise, 41 hangers, a deck spacing of 11.35 m, and an arch-girder weight of 360.3 × 10^3^ kg. The final optimized configuration corresponded to a 23.4 m arch rise, 31 hangers, a 6.0 m deck spacing, and an arch-girder weight of 321.6 × 10^3^ kg. Thus, relative to the reference configuration, the optimization reduced the arch-girder weight by 10.7%. The maximum ULS utilization ratio remained close to 0.99, and the governing location was consistently observed near 0.98 L, indicating that the weight reduction was achieved without relaxing the adopted code-based verification criteria.

In this study, the reference configuration was treated as the baseline traditional design scheme. Its global geometry was based on the available dimensions of the Father Bernatek Footbridge in Krakow and supplemented by the authors’ own field inventory measurements. The numerical model should not be interpreted as a direct reconstruction of the existing bridge, because selected material and cross-section assumptions were adapted to the objective of the present study. The existing footbridge was used mainly as a geometrical and architectural reference, whereas the analysis focused on the code-based material optimization of a steel tubular arch girder. A broader comparison with several independently developed traditional bridge variants was not performed, because tied-arch footbridges with distinctive architectural geometry are project-specific structures and the preparation of alternative complete designs would require separate modeling, sizing, verification, and constructability assessment procedures.

**Figure 13:**
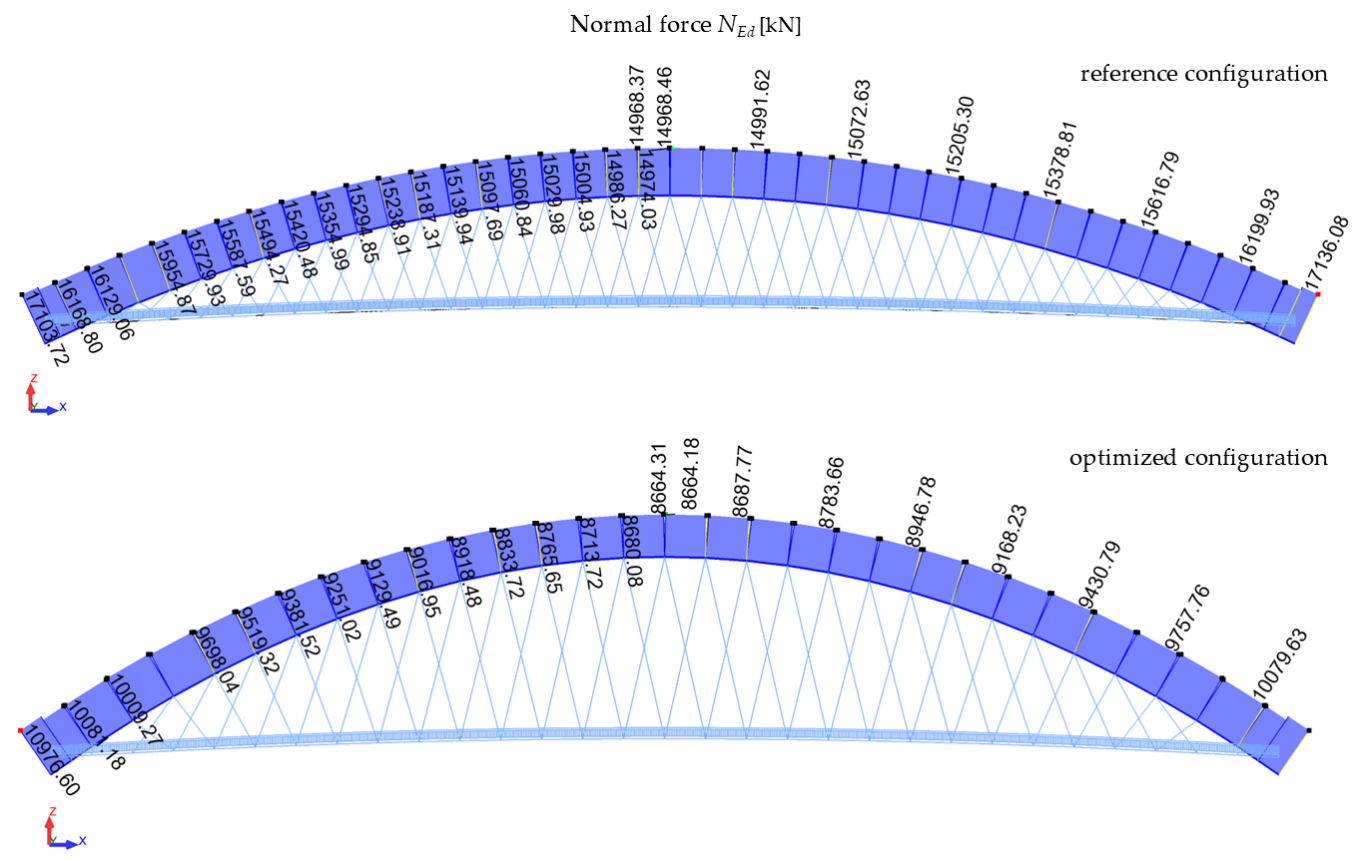
Normal force *N_Ed_* distribution in the arch girder for the reference and optimized configurations under the governing load combination [kN].

**Figure 14:**
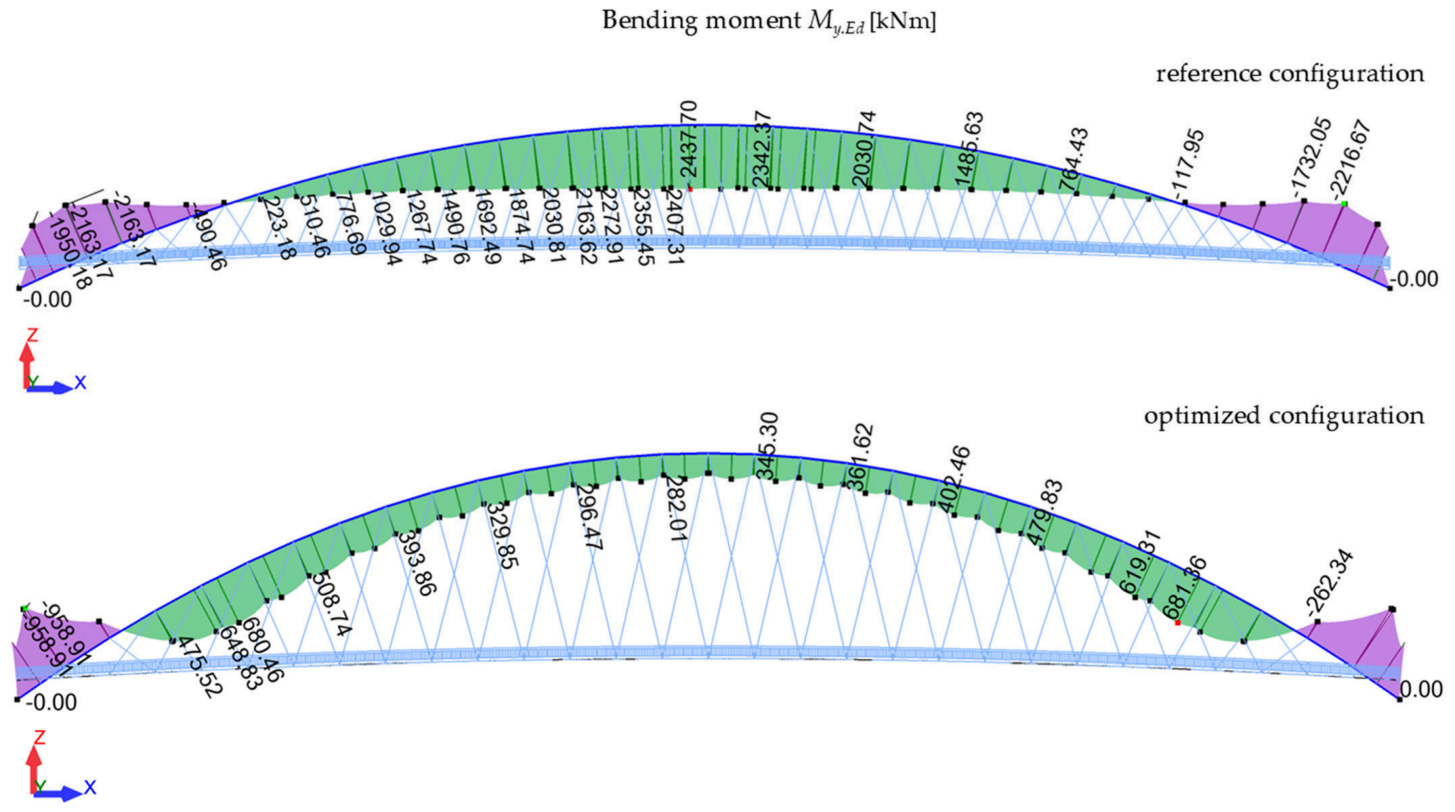
Bending moment *M_y,Ed_* distribution in the arch for the reference and optimized configurations under the governing load combination [kNm].

**Figure 15:**
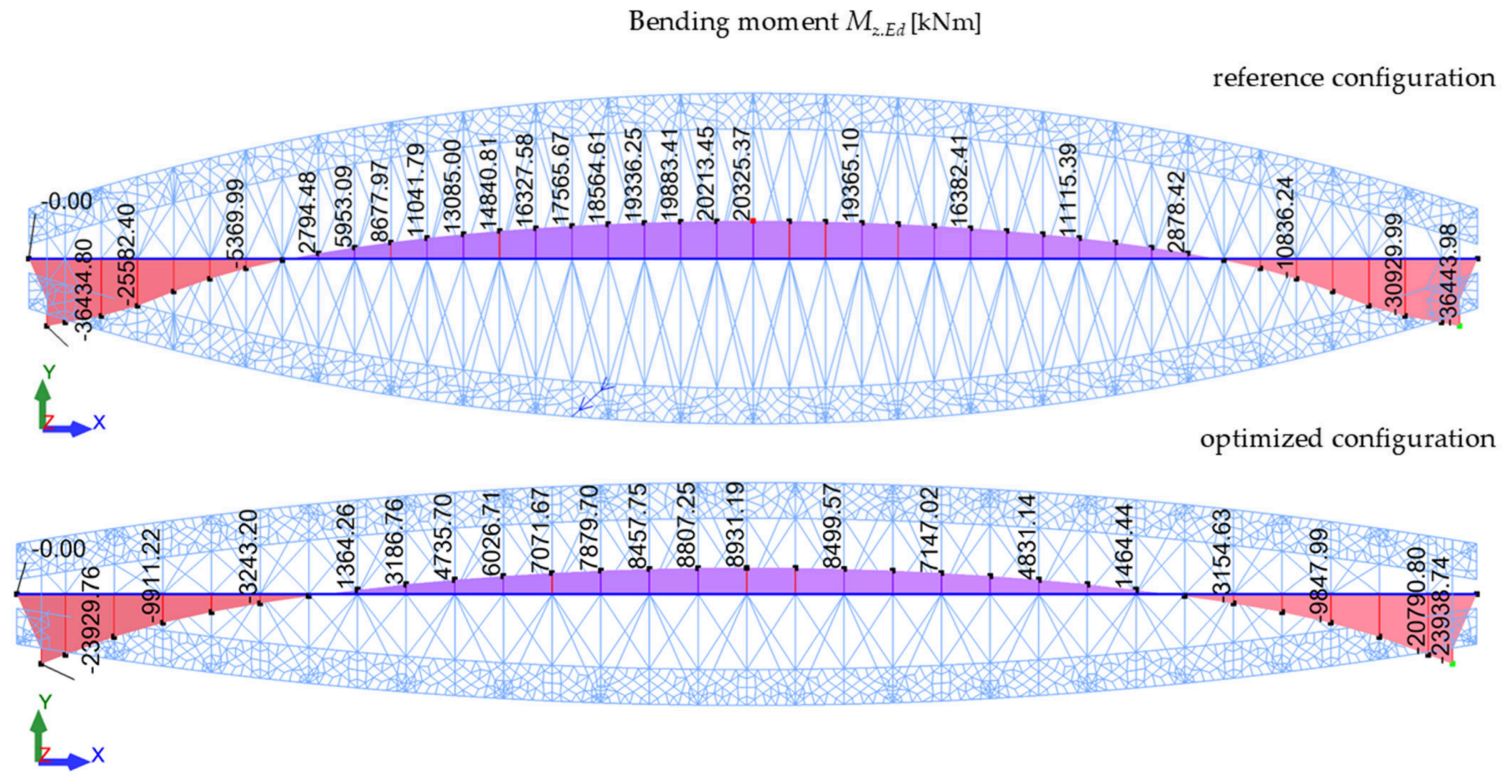
Bending moment *Mz,Ed* distribution in the arch girder for the reference and optimized configurations under the governing load combination [kNm].

Figure 13, Figure 14 and Figure 15 compare the internal force distributions in the arch girder for the reference and optimized configurations under the governing load combination.

The normal force diagram in Figure 13 confirms that, in both configurations, the arch rib works predominantly in compression along its entire length. For the optimized configuration, the compressive force remained high over the full arch, with values generally in the order of 8.0 × 10^3^–1.1 × 10^4^ kN, and the largest compressive forces occurring near the end regions of the arch. The comparison with the reference configuration shows that the optimization did not change the fundamental load-bearing mechanism of the tied-arch system, but modified the geometry and hanger arrangement in a way that enabled a lighter arch cross-section to satisfy the same code-based verification requirements. This confirms that axial compression remained the dominant internal force component, while the optimized configuration achieved a more material-efficient force–section balance.

The bending moment diagrams in Figure 14 and Figure 15 show that the response about the two principal axes was different in both configurations. The bending moment *M_y,Ed_* remained comparatively small over most of the arch, whereas *M_z,Ed_* was the dominant bending component, with the largest values concentrated near the deck-to-arch connection and support regions. Locally increased values of both bending moments were observed in the zone where the suspended decks are connected to the arch girder. This region also corresponds to the location where other structural members are connected to the arch and where local strengthening is provided. Therefore, the local concentration of bending moments and transverse forces in this zone should be interpreted as a connection-related effect rather than as a change in the global load-bearing mechanism of the arch.

Shear forces in both principal directions were also present in the arch girder; however, they were not included in the governing cross-section verification because the decisive Eurocode resistance condition was controlled by the interaction of axial compression and bending moments. Outside the local connection zone, the maximum shear force in the *z* direction *V_z_*_,*Ed*_ was approximately 421 kN. The shear force in the *y* direction reached a local peak of approximately 11,591 kN in the deck-to-arch connection zone, where the arch is connected to the suspended decks and locally strengthened. Outside this connection region, the maximum *V_y_*_,*Ed*_ value was approximately 1994 kN, and the shear force decreased markedly toward the central part of the span. Since the shear response was secondary with respect to the governing axial-compression and bending interaction, and because the largest shear value was a local connection-zone effect, separate shear-force diagrams were not included in the main text.

The comparison between the reference and optimized configurations indicates that the final solution was not obtained by relaxing the structural demand, but by redistributing internal forces through a coordinated change in arch rise, hanger number, deck spacing, and discrete tubular section selection. As a result, the optimized arch girder maintained the same governing structural mechanism—combined axial compression and bending about the z–z axis—while reducing the arch-girder weight from 360.3 × 10^3^ kg to 321.6 × 10^3^ kg. Therefore, the internal force diagrams support the conclusion that the optimization improved material efficiency without compromising the governing ULS behavior of the arch girder.

Overall, the results identify the governing role of the arch rise and the number of hangers in controlling the self-weight of the load-bearing arch. By contrast, the remaining geometric parameters, including deck spacing, deck width, vertical deck curvature, and the vertical position of the abutments relative to the deck, had a secondary influence on the final solution. The minimum-weight configuration obtained in this study corresponded to an arch rise of 23.4 m, 31 hangers, a deck spacing of 6.0 m, and a bridge span of 143 m. These findings confirm that a substantial reduction in arch element self-weight can be achieved by coordinated adjustment of the principal geometric variables while maintaining compliance with the adopted design criteria.

## 4. Discussion

The results indicate that the minimum arch-girder weight was controlled primarily by the interaction between arch rise and hanger arrangement, whereas the remaining geometric variables played a secondary role. This interpretation is consistent with earlier optimization studies of tied-arch and arch bridges, in which minimum-weight solutions were also governed by a limited number of dominant variables under structural constraints [6,7]. In contrast, the present study addresses the optimization problem at the level of a code-verified steel tied-arch footbridge model by directly combining parametric modeling, finite element analysis, and Eurocode-based verification. The optimum did not arise from a uniform contribution of all parameters, but from the combined effect of geometry, force redistribution, and code-based section requirements.

The dominant role of arch rise agrees with previous research showing that geometric proportions strongly affect the response of arch systems. Nazmy [52] showed that rise-to-span ratio is one of the key design parameters influencing both buckling resistance and load-carrying capacity of long-span steel arch bridges. Analytical work by Cai et al. [53] further demonstrated that the geometry of fixed parabolic arches directly affects nonlinear in-plane stability and the transition between buckling modes. From this perspective, the optimum arch rise obtained in the present study should be interpreted as the point at which structural efficiency and section demand are most favorably balanced, rather than as a purely geometric minimum.

The number of hangers was identified as the second governing variable. This finding shows that hanger density should not be treated solely as a detailing decision, because it directly controls the spacing of load-introduction points and therefore the mechanism of force transfer from the decks to the arch girder. A similar broader conclusion was reported by Feng et al. [54], who showed that the optimum design of a steel arch bridge depended on the interaction of several structural variables within a global search framework. In the present work, the favorable hanger range shifted with arch rise, which confirms that the optimum hanger arrangement is geometry-dependent rather than universal.

An important feature of the present results is the stepwise variation in arch self-weight. This behavior is directly related to the adopted discrete section-selection procedure. The arch diameter and wall thickness were not varied as continuous mathematical quantities, but were selected from a set of nominal tubular sections defined by 50 mm diameter increments and 5 mm wall-thickness increments. Such an assumption is consistent with practical preliminary design, in which large circular steel arch ribs are selected or fabricated using nominal dimensions rather than arbitrary continuously varying values. This is particularly relevant for the analyzed bridge, where the load-bearing arch has a span of 143 m, an optimized rise of 23.4 m, and a tubular cross-section with a diameter of approximately 2.45 m. At this scale, the member is closer to a project-specific fabricated steel component than to a small catalog profile.

European standards support this interpretation by defining dimensional ranges and tolerances rather than a continuous set of economically equivalent sections. EN 10210-2:2019 provides dimensional rules for circular hollow sections up to 2500 mm outside diameter and 120 mm wall thickness [50], while EN 10029:2010 covers hot-rolled plates from 3 mm to 400 mm thickness [51]. In the relevant plate-thickness range, EN 10029 tolerances are already on the order of millimeters: −0.8/+1.4 mm for 25–40 mm plates and −1.0/+1.8 mm for 40–80 mm plates in Class A, or ±1.1 mm and ±1.4 mm, respectively, in Class D [51]. Therefore, a much finer numerical variation in wall thickness, for example by tenths of a millimeter, would not be meaningful for preliminary design and would create an artificial impression of precision that is not reflected in practical fabrication.

As a result, small changes in arch rise or hanger number did not necessarily produce a smooth change in steel weight. In several neighboring design variants, the same nominal tubular section was required to satisfy the ULS verification, and the arch weight therefore remained almost constant. A sudden reduction occurred only when a given geometric configuration allowed the next lighter nominal section to satisfy the Eurocode resistance check. Conversely, if the utilization exceeded the allowable limit, a heavier nominal section had to be selected, producing an abrupt increase in self-weight. The observed stepwise curves should therefore be interpreted as a consequence of practical discrete steel section selection combined with code-based verification, rather than as numerical noise or insufficient convergence of the optimization procedure. Similar observations were reported in visual-programming and genetic algorithm (GA)-based optimization studies of arch bridges, where automated search improved design-space exploration, but the final solution remained constrained by feasible member sizing and structural checks [12,54]. This explains why a semi-automated, non-gradient-based search was well suited to the present problem.

The influence of the adopted buckling coefficients should also be interpreted in relation to this threshold-based section selection. The in-plane and out-of-plane buckling factors affect the effective buckling lengths and the corresponding reduction factors used in the Eurocode-based ULS verification of the arch girder. Consequently, their variation may shift the limit at which a heavier or lighter tubular section becomes necessary. However, the sensitivity study showed that, within the final refinement range around the optimum, the in-plane buckling coefficient remained practically constant, while the out-of-plane coefficient changed only slightly. Therefore, the local influence of buckling-factor variation on the final section selection was secondary compared with the effects of arch rise, hanger arrangement, force redistribution, ULS utilization, and discrete tubular cross-section changes. This confirms that the final weight reduction was governed mainly by the coupled geometry–force–section interaction, while the buckling coefficients contributed through the code-based verification threshold.

The limited influence of deck spacing should be interpreted more narrowly. Within the investigated range, increasing deck spacing increased the utilization ratio of the arch member and moved the design away from the optimum. However, this does not imply that deck-related variables are generally unimportant in arch bridges. Nazmy [52] showed that deck position, girder stiffness, bracing stiffness, and support conditions can significantly affect bridge strength and stability. Therefore, the present conclusion should be limited to the investigated tied-arch footbridge and the adopted parameter range: in this structural configuration, deck spacing was less influential than arch rise and hanger arrangement.

From a methodological standpoint, this study confirms the practical value of linking parametric modeling with direct structural verification. Previous work on visual-programming-based optimization demonstrated that combining parametric geometry generation, FEM analysis, and optimization algorithms can support automated studies of arch bridges [12]. The contribution of the present study is that the iterative loop was coupled directly with Eurocode-based ULS and SLS checks, so the objective function was the self-weight of a structurally verified load-bearing arch rather than a simplified surrogate measure. An additional methodological advantage of the workflow is that the governing design response was not assessed on the basis of a single simplified load case. Instead, each candidate geometry was evaluated against more than 600 Eurocode-compliant ULS and SLS load combinations, including different leading and accompanying variable actions, partial and combination factors, asymmetric deck loading, snow load, and wind directions. This broad load-combination set made it possible to identify the governing verification scenario automatically and reduced the risk of underestimating the structural demand caused by manually selected or overly simplified loading assumptions. This makes the proposed workflow especially relevant to preliminary bridge design, where multiple feasible variants must be compared efficiently.

The methodological contribution of this study should therefore be interpreted in terms of process integration and structural interpretation rather than as a direct translation of Eurocode provisions into an algorithm. Eurocode-based verification forms only the constraint-checking module of the workflow. The research contribution lies in coupling this module with parametric geometry generation, FEM model creation, automated load and load-combination definition, nonlinear structural analysis, and iterative section adjustment. This coupling made it possible to observe how changes in arch rise and hanger number modified force redistribution in the arch girder and how these changes interacted with discrete tubular cross-section updates required by ULS verification. Thus, the obtained optimum was not a direct consequence of applying a code equation, but the result of a coupled geometry–force–section interaction within the explored design domain.

Another important feature of the proposed procedure is its interpretability. The progressive domain-reduction strategy allowed the design space to be narrowed in a controlled manner and made it possible to identify the role of individual variables, particularly arch rise and hanger number. This is relevant because the analyzed objective function was not smooth; it changed stepwise due to discrete cross-section updates required by code-based verification. In such a case, the ability to understand why a given design becomes lighter or heavier is as important as the numerical value of the final optimum. The proposed workflow therefore provides a practical link between optimization results and engineering decision-making.

The transferability of the workflow follows from its modular structure. The same sequence—parameter definition, geometry generation, FEM model creation, load assignment, structural analysis, code verification, and response extraction—can be retained for other structural systems. For steel arch bridges, the hanger arrangement, bracing layout, and arch cross-section families could be modified. For CFST arch bridges, the verification module could be extended to include composite steel–concrete interaction and confinement effects. For cable-supported bridges, the procedure could be adapted by introducing cable-force tuning and construction-stage constraints. For roof structures or spatial frames, the same approach could be used to study geometry-sensitive optimization problems governed by stability, deflection, or member utilization. Thus, the proposed method provides a reusable framework for code-based parametric optimization, provided that the structural typology, load model, and design checks are appropriately redefined.

The adopted progressive domain-reduction search should be interpreted as a problem-tailored search strategy rather than as a universal global optimization algorithm. This statement refers only to the selected search strategy and not to the transferability of the proposed computational workflow. The search strategy was selected because the present problem involved a limited number of governing variables, expensive FEM-based evaluations, and a stepwise objective function caused by discrete cross-section changes. By contrast, the workflow itself remains modular: the sequence of parametric model generation, FEM analysis, load-combination evaluation, code-based verification, and response extraction can be adapted to other structural systems after redefining the design variables, load models, member families, and verification criteria. This aspect is particularly important because the objective function did not vary smoothly over the entire design domain. Changes in the required tube diameter or wall thickness produced stepwise variations in the arch self-weight, which reduced the usefulness of optimization procedures that rely on smooth response surfaces or reliable gradient information.

A concise comparison between the adopted progressive search strategy and selected mainstream optimization methods is presented in Table 4. Genetic algorithms, originally developed within the broader framework of adaptation and evolutionary search [55,56], are widely used for nonlinear and discrete optimization problems. Particle swarm optimization [57] and simulated annealing [58] provide alternative derivative-free search strategies, while gradient-based methods [59] and surrogate-assisted approaches [60,61] represent other established optimization frameworks. In the present study, these methods were considered mainly in relation to the high cost of full FEM-based evaluations, the limited number of governing design variables, and the discontinuous character of the section-dependent objective function.

As shown in Table 4, the adopted search strategy was selected for the specific characteristics of the present problem: a limited number of governing variables, expensive FEM-based evaluations, and a stepwise objective function caused by discrete section changes. Therefore, the strategy should not be interpreted as a replacement for general-purpose optimization algorithms. However, the proposed computational workflow is transferable, because the same sequence of parametric geometry generation, FEM model creation, load assignment, structural analysis, code verification, and response extraction can be retained for other bridge or structural typologies. Its main advantage in the present workflow was that it allowed the design domain to be narrowed while preserving direct verification of each analyzed variant. The comparison also indicates that population-based or surrogate-assisted methods may become more advantageous in extended studies involving additional variables, alternative cross-sections, bracing systems, construction stages, or multi-objective criteria. Therefore, a direct benchmark against these methods is recommended as a future extension of the proposed workflow.

The following limitations define the scope within which the obtained optimum should be interpreted and indicate the main directions for future development of the workflow. First, the optimization was restricted to a single primary objective, namely the minimization of the load-bearing arch girder self-weight. Although the deck system, bracing, railing, and secondary members were included in the FEM model, participated in load transfer, and were checked according to the applicable ULS and SLS requirements, they were not independently optimized as separate subsystems. Therefore, the obtained 10.7% arch-girder weight reduction relative to the reference configuration should be interpreted as an arch-girder optimum within the analyzed tied-arch footbridge model, rather than as a complete full-bridge minimum-mass or minimum-cost solution.

Second, the analysis was limited to one steel tied-arch footbridge typology and to the final service-stage configuration. Construction-stage effects, temporary supports, erection loads, hanger or cable force adjustment, support settlements, and stage-dependent stability were not included in the optimization loop. Similarly, the coupled influence of deck width and deck curvature on arch internal-force redistribution was not treated as a separate multidimensional sensitivity problem. These aspects would require an extended parameter space and additional FEM evaluations and are therefore identified as future extensions of the proposed workflow.

Third, the objective function did not include the mass and cost implications of the hanger system, anchorages, connection details, local stiffening, fabrication, transport, installation, corrosion protection, inspection, or maintenance. Consequently, a configuration that minimizes arch-girder weight may not necessarily be optimal from the perspective of total bridge life-cycle cost. Future work should therefore extend the objective function to include the arch girder, hangers, anchorages, deck girders, bracing, deck elements, and connection details.

A further limitation concerns serviceability beyond static deflection. The present optimization included static ULS/SLS verification and a conservative deflection check, but it did not include pedestrian-induced dynamic response, comfort criteria, modal tuning, fatigue assessment, or human–structure interaction effects. These aspects are important for lightweight footbridges, where mass reduction may affect dynamic response [63,64,65,66]. Future extensions should therefore include modal analysis, vibration serviceability assessment, fatigue verification, and sensitivity analysis with respect to geometric imperfections and modeling assumptions.

Finally, the adopted progressive domain-reduction search should be interpreted as a problem-tailored strategy rather than as a universal global optimization method. Although it was efficient and interpretable for the present problem, future studies should benchmark it against genetic algorithms, particle swarm optimization, simulated annealing, surrogate-assisted optimization, and hybrid approaches for larger design domains and multi-objective formulations. Such extensions should also consider alternative material systems, including CFST arches, high-strength steel members, steel–UHPC composite systems, and other hybrid structural solutions.

From a materials-oriented perspective, the relevance of the present study lies in quantifying how geometric decisions affect the demand for structural steel in a tubular arch girder. The optimization did not treat the cross-section as a continuously variable mathematical parameter, but as a discrete design choice governed by code-based resistance requirements. Consequently, the observed stepwise variation in self-weight reflects the practical relationship between structural form, utilization level, and available steel tube sections. This is important for material-efficient bridge design, because even moderate reductions in member weight may translate into lower steel consumption, reduced fabrication and transport demand, and improved preliminary assessment of alternative material solutions. The same workflow can be extended to compare different material systems, including high-strength steel, CFST arch members, steel–UHPC composite systems, or other hybrid solutions, provided that the corresponding material models and verification criteria are introduced. Thus, the material-related contribution of this study lies in demonstrating how structural form, code-based utilization, and discrete steel section selection jointly determine the demand for structural steel in the arch girder.

Overall, the significance of this study lies not only in the obtained 10.7% reduction in arch-girder weight relative to the reference configuration, but also in identifying the structural mechanism behind that reduction. The optimum resulted from the interaction between arch rise, hanger density, and ULS-controlled section changes, rather than from uniform sensitivity of the full parameter set. This confirms the practical value of code-based parametric optimization for tied-arch footbridges and shows that even a semi-automated workflow can reveal structural dependencies that are difficult to detect through conventional iterative sizing.

Future research should reformulate the present procedure as a multi-objective optimization problem. Possible objective functions include minimization of structural mass, material and fabrication cost, embodied carbon, maximum utilization ratio, deflection, natural-frequency sensitivity, fatigue damage, and construction-stage stress demand. Such a formulation would allow the generation of Pareto-optimal design sets instead of a single minimum-weight solution. This would be particularly useful in preliminary bridge design, where decision-makers must balance structural efficiency, economy, constructability, durability, and architectural requirements. Multi-objective formulations may also help identify whether the arch rise and hanger number remain the dominant variables when additional criteria, such as dynamic comfort or fabrication cost, are included.

Another important direction is the extension of the verification module. In the present study, the final design was governed mainly by ULS utilization and discrete changes in the required tubular cross-section. Future work should include fatigue assessment of hangers and welded details, dynamic analysis under pedestrian loading, and sensitivity analysis with respect to geometric imperfections and modeling assumptions. For CFST arch solutions, the procedure should be expanded to account for composite steel–concrete interaction, confinement effects, construction sequence, and time-dependent material behavior. These extensions would make the workflow more suitable for the design of long-span arch bridges and other complex bridge systems.

## 5. Conclusions

This study developed a semi-automated, API-driven workflow for the code-based parametric optimization of the load-bearing arch girder in a steel tied-arch footbridge by combining parametric modeling, finite element analysis, and Eurocode-based structural verification within a single iterative loop. The proposed procedure enabled systematic evaluation of geometric variants and identification of the minimum-weight solution within the analyzed design domain.

The results showed that the governing variables for arch-girder weight were arch rise and the number of hangers, whereas deck spacing had a secondary and unfavorable effect on structural response. The optimum solution within the investigated domain corresponded to an arch rise of 23.4 m, 31 hangers, and a deck spacing of 6.0 m for a bridge span of 143 m.

The optimization reduced the load-bearing arch-girder weight by 10.7% relative to the reference configuration, from 360.3 × 10^3^ kg to 321.6 × 10^3^ kg. Although this reduction is moderate in absolute terms, it demonstrates that coordinated adjustment of arch geometry and hanger arrangement can reduce structural steel demand while maintaining ULS/SLS compliance.

The results also showed that the final solution was governed by ULS verification and by discrete changes in the required cross-section, which produced a stepwise variation in structural mass across the analyzed parameter range. The optimized configuration should therefore be interpreted as an arch-girder optimum within a complete code-verified bridge model, rather than as a full-bridge minimum-mass or minimum-cost solution. Further system-level optimization should include the mass and cost of hangers, anchorages, main deck girders, bracing, deck elements, and connection details. The same workflow can also be applied to alternative structural steel grades, provided that the corresponding material properties, product availability, cross-section classification, buckling resistance, and Eurocode verification parameters are updated accordingly. Future extensions should therefore include a combined mass–cost assessment of different steel grades, since higher-yield-strength steel may reduce the required cross-section size and self-weight, but may also affect stiffness, natural frequencies, vibration serviceability, and possible resonance effects.

Overall, the proposed workflow can support the preliminary design of tied-arch footbridges by linking parametric modeling directly with automated load-combination evaluation and code-based structural assessment. A direct numerical benchmark against genetic algorithms, particle swarm optimization, and surrogate-assisted optimization methods is recommended as a future extension of the proposed workflow, particularly for larger design domains and multi-objective formulations. Because the workflow is modular, the same methodological structure can be transferred to other geometry-sensitive structural systems by redefining the parametric model, load cases, member families, and design-code verification rules. This makes the proposed approach applicable not only to tied-arch footbridges, but also to other arch, truss, cable-supported, composite, and spatial frame structures.

Future work should extend the proposed workflow in four main directions. First, the optimization should be reformulated as a multi-objective problem including structural mass, cost, embodied carbon, deflection, dynamic comfort, fatigue performance, and member utilization. Second, construction-stage analysis should be incorporated into the iterative loop. Third, the procedure should be benchmarked against genetic algorithms, particle swarm optimization, simulated annealing, surrogate-assisted optimization, and hybrid approaches for larger design domains. Finally, the workflow should be extended to alternative structural systems, particularly CFST arches, cable-supported bridges, truss arch bridges, composite decks, and spatial frame structures. A particularly relevant extension would be the application of the workflow to CFST arch members, including the interaction between the confined concrete core and the surrounding steel tube.

## Figures and Tables

**Table 1:** Variable parameters of the parametric footbridge model and the adopted ranges used in the study.

Parameter	Min [m/pcs]	Max [m/pcs]	Step [m/pcs]	Optimized Value [m/pcs]
Bridge span	140	250	1	1
Arch rise	15	30	0.1	0.1
Vertical position of abutments relative to the deck	−5	0	0.2	0.2
Number of hangers	17	41	2	2
Width of the deck	2	6	0.2	0.2
Horizontal spacing of the decks	0	7	0.1	0.1
Vertical curve height of the decks	0	5	0.1	0.1
Horizontal curve height of the decks	6	17	0.2	0.2
Cross-section diameter of the arch	2	3	0.05	0.05
Cross-section thickness of the arch	0.03	0.045	0.005	0.005

**Table 2:** Structural elements, adopted cross-sections, and steel grade.

Element	Cross-Section [mm]	Steel Grade
Arch rib (purple/violet)	PO 2450 × 35 (optimal)	S 460 M/ML
Hanger (green)	PO 40 (optimal)	
X bracing (purple/magenta)	RO 219.1 × 12	
Stringer beam (brown)	IPE 330	
Deck panels (light grey)	PL 20	
Crossbeam (light purple)	RO 508 × 16	
Handrail (blue-grey)	RO 219.1 × 5	
Balustrade posts (green)	RP 100 × 50 × 5	
Longitudinal infill members of the balustrade (purple/violet)	RP 80 × 40 × 5	
Vertical infill members of the balustrade (light green)	RO 42.4 × 2.6	

Note: The colors in parentheses correspond to the graphical representation of the structural components in Figure 5.

**Table 3:** Evolution of the main arch-girder parameters during the optimization procedure.

Parameter	Reference Configuration	Optimization Stage				
		1	2	3	4	5 (Optimum)
Bridge span [m]	143	143	143	143	143	143
Arch rise [m]	15	22.5	22.5	24.0	20.0	23.4
Number of hangers [-]	41	29	29	27	25	31
Deck spacing [m]	11.35	6.0	5.5	6.0	6.0	6.0
Arch cross-section [mm]	PO 2850 × 35	PO 2500 × 35	PO 2500 × 35	PO 2450 × 35	PO 2500 × 35	PO 2450 × 35
Arch-girder weight [kg]	360.3 × 10^3^	326.7 × 10^3^	326.7 × 10^3^	322.7 × 10^3^	322.6 × 10^3^	321.6 × 10^3^
Arch-girder weight reduction						
stage/cumulative [%]/[%]	-	9.3/9.3	0/9.3	1.1/10.4	0.03/10.43	0.3/10.7
Maximum ULS utilization ratio [-]	0.99	0.95	0.94	0.99	0.99	0.99
Governing ULS location [-]	≈0.98 *L*	≈0.98 *L*	≈0.98 *L*	≈0.98 *L*	≈0.98 *L*	≈0.98 *L*
Governing load combination [-]	185 SGN/176	185 SGN/176	185 SGN/176	185 SGN/176	185 SGN/176	185 SGN/176
Maximum SLS deflection ratio [-]	0.59	0.88	0.82	0.94	0.89	0.94
SLS limit [-]	*L*/350	*L*/350	*L*/350	*L*/350	*L*/350	*L*/350

**Table 4:** Comparison of the adopted progressive search method with selected mainstream optimization approaches.

Optimization Method	Advantages and Limitations in the Present Study	Decision	Reason
Progressive domain-reduction search	Transparent, deterministic, and computationally efficient for a limited number of governing variables; no formal guarantee of global optimum.	Adopted	It provided sufficient exploration of the relevant design domain with a limited number of full FEM evaluations.
Genetic algorithm	Strong global-search capability for nonlinear and discrete problems, but many FEM evaluations are required [55,56].	Not adopted	The expected number of full FEM-based evaluations was too high for the current workflow.
Particle swarm optimization	Suitable for nonlinear continuous variables, but requires many particles and parameter tuning [57].	Not adopted	The problem was governed by a limited number of dominant variables, which favoured a more controlled deterministic search.
Simulated annealing	Can escape local minima, but convergence depends on cooling and neighbourhood settings [58].	Not adopted	Local refinement after domain reduction was more direct for the observed stepwise response.
Gradient-based optimization	Efficient for smooth problems, but unsuitable for stepwise section-dependent objective functions [59].	Rejected	Discrete changes in tube diameter and wall thickness made the objective function non-smooth.
Surrogate-based optimization	Reduces simulation cost after model training, but requires additional sampling and validation [60,61].	Reserved for future work	Direct Eurocode-based verification of each design variant was prioritized in the current study.
Exhaustive grid search	Robust but inefficient for broad discretized spaces [62].	Used only locally	Full enumeration of the original design domain was computationally inefficient, but local grid refinement was useful after narrowing the promising region.

Note: FEM, finite element method. The comparison refers to the present optimization problem, in which each design evaluation required parametric model generation, FEM analysis, load-combination evaluation, and Eurocode-based ULS/SLS verification. The methods not adopted in this study were not considered generally unsuitable, but less efficient for the current design task because of the high cost of repeated FEM evaluations, the limited number of governing variables, and the stepwise objective function resulting from discrete cross-section changes.

## Data Availability

The original contributions presented in this study are included in the article. Further inquiries can be directed to the corresponding author.

## Abbreviations

The following abbreviations are used in this manuscript:

FEM	Finite Element Method
ULS	Ultimate Limit State
SLS	Serviceability Limit State
UHPC	Ultra-High-Performance Concrete
CFST	Concrete-Filled Steel Tube
API	Application Programming Interface
GA	Genetic Algorithm

## Appendix A

### Python Script—Tension-Only Bars

# Load the Python Standard and DesignScript Libraries
import sys
import clr
import time
clr.AddReference(‘ProtoGeometry’)
from Autodesk.DesignScript.Geometry import *
from System import Environment
user = Environment.GetFolderPath(Environment.SpecialFolder.ApplicationData)
clr.AddReference(user + r“\Dynamo\Dynamo Revit\3.3\packages\Structural Analysis for Dynamo\bin\interop.RobotOM.dll”)
from RobotOM import *
from System import Object
# The inputs to this node will be stored as a list in the IN variables.
dataEnteringNode = IN
# The inputs to this node will be stored as a list in the IN variables.
lista = IN [0]
app = RobotApplicationClass()
# Place your code below this line
idstring = “”
for i in lista:
   idstring += str(i)
   idstring += “ ”
Structure = app.Project.Structure
bars = Structure.Bars
bSel = Structure.Selections.Create(1)
bSel.FromText(idstring)
bars.SetTensionCompression(bSel, 1)
# Assign your output to the OUT variable.
OUT = 1

## Appendix B

### Python Script—Simulate Wind

# Load the Python Standard and DesignScript Libraries
import sys
import clr
clr.AddReference(‘ProtoGeometry’)
from Autodesk.DesignScript.Geometry import *
from System import Environment
user = Environment.GetFolderPath(Environment.SpecialFolder.ApplicationData)
clr.AddReference(user + r“\Dynamo\Dynamo Revit\3.3\packages\Structural Analysis for Dynamo\bin\interop.RobotOM.dll”)
from RobotOM import *
from System import Object
# The inputs to this node will be stored as a list in the IN variables.
lista = IN [0]
app = RobotApplicationClass()
# Place your code below this line
structure = app.Project.Structure
loadCases = structure.Cases
wind = loadCases.WindLoadsSimulationEngine
windParams = wind.Params
windParams.DeviationPercent = 1
windParams.DirectionXNEnabled = False
windParams.DirectionXNYNEnabled = False
windParams.DirectionXNYPEnabled = False
windParams.DirectionXPEnabled = True
windParams.DirectionXPYNEnabled = False
windParams.DirectionXPYPEnabled = True
windParams.DirectionYNEnabled = False
windParams.DirectionYPEnabled = True
windParams.OpeningsClosed = True
windParams.TerrainLevel = *−*2
windParams.Velocity = 22
wind.Generate()
# Assign your output to the OUT variable.
OUT = 1
